# Tissue engineered in-vitro vascular patch fabrication using hybrid 3D printing and electrospinning

**DOI:** 10.1016/j.mtbio.2022.100252

**Published:** 2022-04-14

**Authors:** Isabel Mayoral, Elisa Bevilacqua, Gorka Gómez, Abdelkrim Hmadcha, Ignacio González-Loscertales, Esther Reina, Julio Sotelo, Antonia Domínguez, Pedro Pérez-Alcántara, Younes Smani, Patricia González-Puertas, Ana Mendez, Sergio Uribe, Tarik Smani, Antonio Ordoñez, Israel Valverde

**Affiliations:** a Cardiovascular Pathophysiology Group, Institute of Biomedicine of Seville- IBiS, University of Seville /HUVR/CSIC, Seville, Spain; b Advanced Therapies and Regenerative Medicine Research Group.General Hospital, Alicante Institute for Health and Biomedical Research (ISABIAL), Alicante, Spain; c Department of Molecular Biology and Biochemical Engineering, Universidad Pablo de Olavide, Seville, Spain; d Department Mechanical, Thermal and Fluids Engineering, School of Engineering, University of Málaga, Málaga, Spain; e Department of Mechanical and Manufacturing Engineering, University of Seville, Seville, Spain; f School of Biomedical Engineering, Universidad de Valparaíso, Valparaíso, Chile; g Millennium Institute for Intelligent Healthcare Engineering, iHEALTH, Millennium Nucleus in Cardiovascular Magnetic Resonance, Cardio MR, and Biomedical Imaging Center, Pontificia Universidad Católica de Chile, Santiago, Chile; h Doxa Microfluidics, SL, Malaga, Spain; i Department of Molecular Biology and Biochemical Engineering, Andalusian Center of Developmental Biology, CSIC, University of Pablo de Olavide, Seville, Spain; j Pediatric Cardiology Unit, Hospital Virgen Del Rocio, Seville, Spain; k Radiology Department, School of Medicine, Pontificia Universidad Católica de Chile, Santiago, Chile; l Department of Medical Physiology and Biophysics, School of Medicine, University of Seville, Seville, Spain; m School of Biomedical Engineering and Imaging Sciences, King's College London, London, United Kingdom; n Department of Pharmacology, Pediatric and Radiology, School of Medicine, University of Seville, Seville, Spain

**Keywords:** Tissue engineering, Vascular graft, 3D printing, Electrospinning, Mesenchymal stem cells, Three-dimensional, 3D, tissue engineering vascular grafts, TEVG, fused deposition modelling, FDM, vascular smooth muscle cells, VSMC, mesenchymal stem cells, MSC, derived VSMC, dVSMC, extracellular matrix, ECM, computed tomography, CT, computation fluid dynamic, CFD, wall shear stress, WSS, transforming growth factor beta 1, TGFβ-1, platelet-derived growth factor composed by two beta chains, PDGF-BB, bone morphogenetic protein, BMP4, room temperature, RT, western blotting, WB, Reverse Transcription, Rt, anti-alpha-smooth muscle actin, α-SMA, anti-smooth muscle protein 22, SM-22, anti-fibroblast specific protein 1, FSP1, anti-cluster of differentiation 31, CD31, endothelin-1, ET-1, Endothelin Receptor A, ETA, Endothelin Receptor B, ETB

## Abstract

Three-dimensional (3D) engineered cardiovascular tissues have shown great promise to replace damaged structures. Specifically, tissue engineering vascular grafts (TEVG) have the potential to replace biological and synthetic grafts. We aimed to design an in-vitro patient-specific patch based on a hybrid 3D print combined with vascular smooth muscle cells (VSMC) differentiation. Based on the medical images of a 2 months-old girl with aortic arch hypoplasia and using computational modelling, we evaluated the most hemodynamically efficient aortic patch surgical repair. Using the designed 3D patch geometry, the scaffold was printed using a hybrid fused deposition modelling (FDM) and electrospinning techniques. The scaffold was seeded with multipotent mesenchymal stem cells (MSC) for later maturation to derived VSMC (dVSMC). The graft showed adequate resistance to physiological aortic pressure (burst pressure 101 ​± ​15 ​mmHg) and a porosity gradient ranging from 80 to 10 ​μm allowing cells to infiltrate through the entire thickness of the patch. The bio-scaffolds showed good cell viability at days 4 and 12 and adequate functional vasoactive response to endothelin-1. In summary, we have shown that our method of generating patient-specific patch shows adequate hemodynamic profile, mechanical properties, dVSMC infiltration, viability and functionality. This innovative 3D biotechnology has the potential for broad application in regenerative medicine and potentially in heart disease prevention.

## Highlights

• This study combines multidisciplinary approach for bioprinting patient-specific.
• We create a 3D scaffold, printed using a hybrid fused deposition modelling and electrospinning techniques.
• The graft shows adequate resistance to physiological aortic pressure and a porosity gradient.
• Multipotent mesenchymal stem cells seeded in the scaffold are differentiated to derived vascular smooth muscle cells.
• dVSMC shows adequate endothelin- 1 induced Ca2+ increase associated with ETA overexpression.

## Graphical abstract

**Figure:**
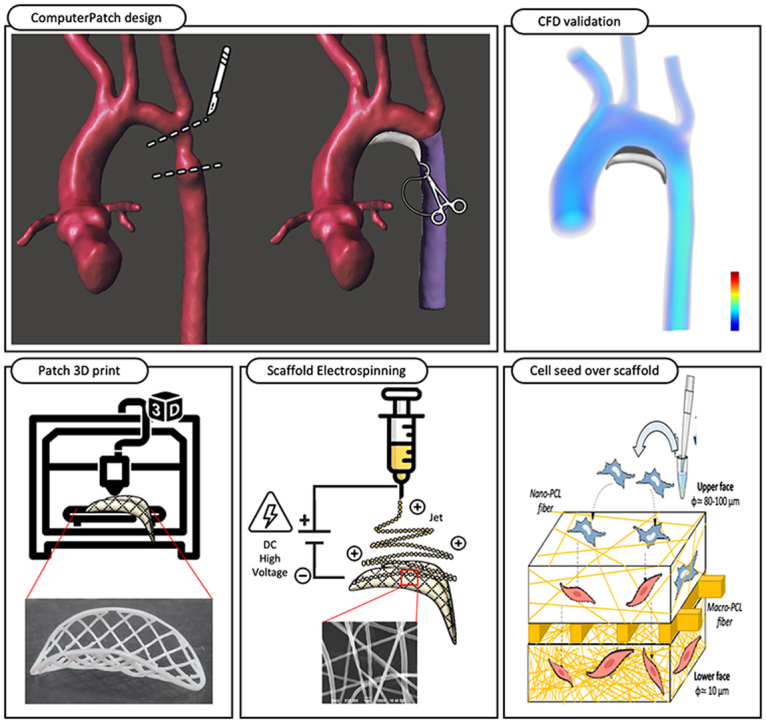

## 1 Introduction

Surgical repair of aortic arch is one of the most technically challenging procedures in children with congenital heart disease, particularly in the case of sever aortic arch hypoplasia, coarctation and Norwood procedure [1]. Several technical modifications have been introduced in recent years, including the use of vascular grafting, to avoid the high rates of recoarctation (11–37%) [2], improving long-term outcomes. Some factors have been identified that may contribute to high rates of recoarctation such as Hand-crafted patch relying on the surgeon's expertise to ensure that its geometry adapts to the arch curvature and guarantee laminar flow. Although arch continuity may be satisfactorily restored, unnoticed turbulent flow may be generated resulting in increased afterload and decreased ejection fraction [3]. Properties of the graft is another key element. Homografts and synthetic grafts inevitably suffer from calcification, immune reaction, inflammation and infection [[4], [5], [6], [7]]. But most importantly, these grafts do not grow with patient growth which makes them especially unsuitable for the pediatric population [8]. This results in deshiscence, thrombosis, bleeding, reoperations, increased morbidity and mortality [[1], [2], [3]].

Tissue engineering is an emerging field which combines biology, medicine and engineering yielding the promise of fabricating long-lasting vascular patches [[9], [10], [11], [12], [13], [14]]. 3D printing offers the possibility of engineering biological active multilayer grafts combining cells and biomaterials mimicking the interstitial matrix structure [12,15]. Three-dimensional printing strategies for tissue engineering vascular graft (TEVG) can be mainly divided into scaffold-base approach (3D printing scaffold and cell culture) and bioprinting approach (incorporation of cells 3D printing material) [16,17]. Some critical aspects of bioprinting are the selection of biocompatible polymers, low printing resolution as well as low speed and high fabrication cost. This results in low efficiency, limited cell density due to high cell destruction, low porosity leading to limited nutrient diffusion and limited creation of vascular networks [16,17].

The scaffold-base approach may offer some advantages over 3D bioprinting [18]. It is based on the assumption that cells seeded over biocompatible scaffolds would serve as a site of cell attachment, migration and growth resulting in the formation of a neotissue with a pre-defined geometry. There are several fabrication methods applied in scaffold fabrication such as extrusion, inkjet and light-based systems [[19], [20], [21]]. Among available techniques, hybrid fused deposition modelling and electrospinning may be one of the most promising methods [[22], [23], [24]]. Fused deposition 3D printing provides a robust scaffold with predefined geometry while electrospinning provides small fiber diameters which resemble the native extracellular matrix (ECM) and offer a large surface to volume ratio [22]. Natural and synthetic polymers can be used for 3D printing either as polymers or as hydrogels including propylene fumarate, polyurethane, polytetrahydrofuran diacrylate, alginate, silicone and polycaprolactone [15]. Polycaprolactone (PCL) is a synthetic polymer which has shown great potential for TEVG due to its cytocompatibility and mechanical properties [25]. Due to its biodegradability, the scaffold has the potential to be later replaced by native interstitial ECM.

Once the scaffold structure is fabricated, cells are seeded and grown on printed structures. One of the current limitations is the shortage of endothelial and muscle cell availability [26]. However, stem cells have great potential to provide large numbers of cells with an effective differentiation capacity [27]. Mesenchymal stem cells (MSC) can differentiate towards differentiated vascular smooth muscle cells (dVSMC) lineages and so could be used for TEVG [28]. Over time the cells will replace the polymer with their own interstitial matrix, ensuring the integrity and mechanical properties of the patch [24]. The final result will be a patch entirely composed of autologous tissue in the absence of any synthetic material.

Previous research studies focused on combining 3D printing and electrospinning to create bio-tubular vascular scaffolds [29]. However, these tubular scaffolds are generic, not adapted to patient-specific anatomy and importantly some of the cardiovascular surgical techniques in children require curved patches rather than conventional tubes. The aim of this study is to combine, as a proof of concept, the principles of computational fluid dynamics, mechanical engineering and tissue engineering to design a personalized vascular patch. Based on a particularly complex individual anatomy. Computational fluid dynamics simulations and virtual surgical planning will be used to design the shape of the vascular patch with the most efficient haemodynamic surgical behaviour. Fused deposition modelling 3D printing will replicate the predefined patch geometry and combined with electrospinning technique will provide a preliminary 3D supportive structure for the cells. Finally, the scaffold will be seeded with multipotent mesenchymal cells for later maduration to derived vascular smooth cells (dVSMC). This may open new doors for future investigations to achieve a patient specific autologous patch with the potential for remodeling, repair, durability and growth, particularly important in the pediatric population.

## 2 Materials and methods

The study methodology is divided in three main sequential steps: (A) Design a patient-specific patch based on patient's medical images, (B) Fabrication of the scaffold using 3D printing and electrospinning and (C) cell culture over the scaffold and cell differentiation (See graphical abstract). The first step is the patch design. It requires the selection of patient-specific medical images and creation of the 3D anatomical geometry for virtual aortic surgery. Two potential surgical reconstructions, adapted to the individual patient anatomy, were computer-designed using two patches of different sizes and curvature shapes. The patches were mathematically compared using computational fluid dynamics. The patch which showed a more hemodynamically efficient geometry was selected for scaffold printing and cell seed. The second stage was transforming the virtual geometry into a physical patch by 3D printing. This was performed initially by printing a gross and solid scaffold by fused deposition modelling and later by adding a fine fiber mesh to recreate the extracellular matrix by electrospinning. Finally, the scaffolds were seeded with pluripotential MSC for latter differentiation towards dVSMC.

### 2.1 Design of patient specific patch

#### 2.1.1 Patient selection and medical imaging

A retrospective case of a two months-old girl and 4.8 ​kg with diagnosis of coarctation of the aorta and transverse arch hypoplasia was selected to create the patch for surgical reconstruction. A prospectively acquired electrocardiography gated cardiac computed tomography (CT) (Somatom Definition Flash, Siemens Healthcare, Erlangen, Germany), spatial resolution 1x1x0.75 ​mm slice thickness was used. A total of 2 ​ml/kg of intravenous contrast (injection rate range 2.0–3.0 ​ml/s) was injected with a saline chaser.

#### 2.1.2 Patch geometric design

**Fig. 1:**
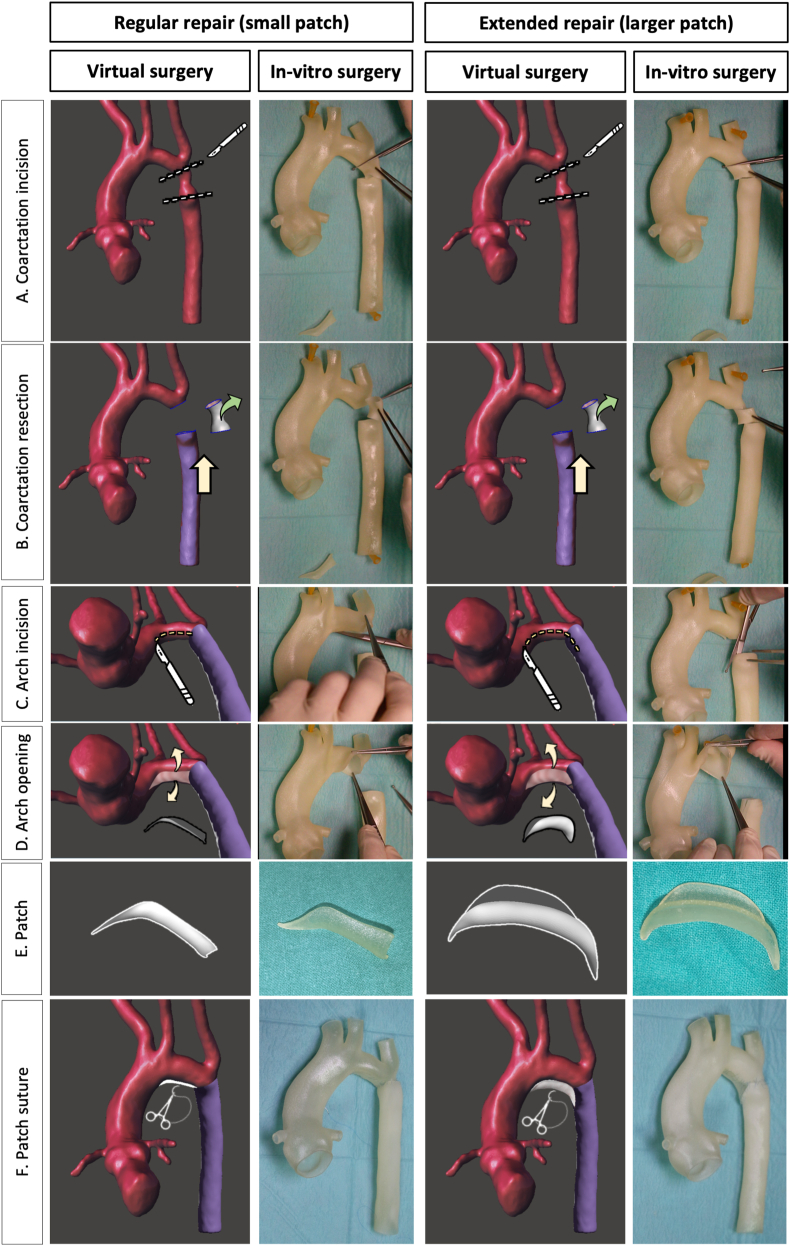
**Virtual and in-vitro surgery plan**. Two types of surgical reconstructions were performed, (A) regular repair with small patch and (B) extended repair with larger patch. **A.** Surgical incision distal to the left subclavian artery and below the aortic isthmus. **B.** Resection of the hypoplastic isthmus. The descending aorta is brought up to the aortic arch. **C.** A longitudinal incision was made exclusively along the undersurface of the aortic arch in the regular repair, but it was lengthened to the ascending and the descending aorta in the extended repair. **D.** The undersurface of the aorta was opened for preparation for reconstruction with the patch. **E.** The small patch used in the regular repair and the larger patch used in the extended repair are shown. **F.** The patch was sutured increasing the section of the aorta.

The process of converting medical images to virtual 3D models was performed according to the previously published methods [[30], [31], [32]]. The CT data were exported in DICOM format and imported into ITK snap (version 3.8.0, University of Pennsylvania, Philadelphia, US). Segmented aortic geometric data were exported as a 3D surface file into Meshmixer version 11.0.544 (Autodesk Inc. US) for computer-aided design. Meshmixer was used to perform computer virtual surgery under the supervision and guidance of one pediatric cardiac surgeon with over 20 years’ experience (Fig. 1, Virtual surgery). In order to find the optimal aortoplasty which would ensure an adequate concordance between the proximal and distal segments of the aorta, two types of surgical reconstruction with two patches of different sizes and shapes were performed: regular and extended repair. A longitudinal incision was made along the undersurface of the aortic arch. This incision was confined to the undersurface of only the aortic arch in the regular repair and prolonged into the ascending and descending aorta in the extended repair. The coarctation segment was excised. Using virtual tools, the aorta was opened while maintaining the same aortic surface area (the aortic wall is not stretched). Two virtual patches were created with different sizes and curvatures to match both the length of the aortic incision and the diameter discrepancy between the ascending and descending aorta. The patches were created with a width that would ensure normal reconstructed transverse arch and descending aortic diameters in accordance with Z-scores [33]. To verify that the virtual surgery process was reproducible in vitro, two replicas of the aortic anatomy along with the two types of patches (regular and extended) were 3D printed. 1 ​mm wall Aortas and patches were printed in a Objet Connex 260V Stratasys 3D printer. Translucent and flexible Agilus material was used. The parts were printed in the hospital Fabrication Laboratory. As shown in Fig. 1 (In–vitro surgery) and supplemental online video 1, we demonstrated that both patch repair solutions were technically feasible.

#### 2.1.3 Computational fluid dynamics

**Fig. 2:**
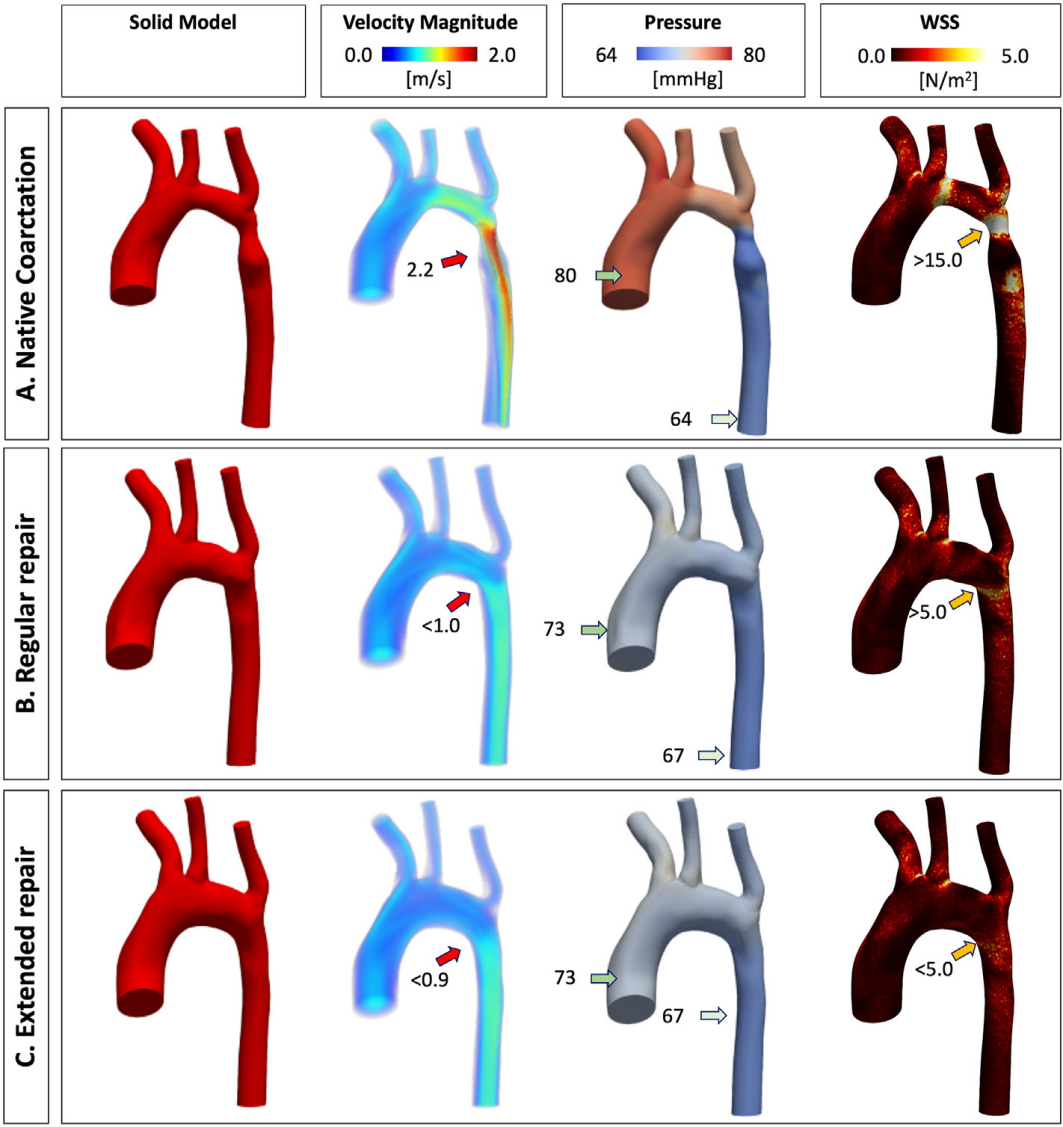
**Computational fluid dynamics steady simulation results.** The simulation is shown in the original state at birth presentation, native coarctation (A) and after the two potential surgical corrections: regular repair (B) and extended repair (C). The first column shows the solid model generated from the STL file of the geometry. The second column shows the velocity magnitude (m/s). The third column shows the pressure (mmHg). The fourth column shows the wall shear stress (WSS) expressed in N/m^2^. The color bar (scale intensity for velocity magnitude, pressure and WSS) is adjusted taking as reference the native aortic coarctation results. The arrows indicate the values in those locations.

Although both surgical repairs were technically feasible, the hemodynamic behavior induced by the patch in the aortic geometry is unknown, for that reason three steady-state computation fluid dynamic (CFD) simulations (one native coarctation and two repaired coarctation) were performed using the custom software CRIMSON (available at: http://www.crimson.software/) (Fig. 2). The objective of CFD simulation was to find the surgical repair that, individualized to the patient, would offer the most optimal hemodynamic conditions, in short computational time, that may be more feasible for diagnostic in clinical routine.

The purpose of these simulations was to visualize how the geometry affects the flow hemodynamics, and if parameters such as wall shear stress (WSS) and pressure were fixed with the correction. We created the solid model for each case using the STL files. The windkessel models for the outflow branches was defined with the procedure described in Xiao et al. [34], the ascending aorta inflow was set at 17.3 ​ml/s for each CFD simulation, the descending aorta outflow was set 8.6 ​ml/s for the Native Coarctation, and 10.3 ​ml/s for each correction using the velocity data of one representative matched patient based on magnetic resonance imaging data, and the initial pressure was set to 60 ​mmHg for all simulations. The simulations were executed in a standard computer (Intel® Core™ i7-10875U 2.3 ​GHz, 16 ​GB Ram, GeForce® RTX 2070), with a time consuming of 60 ​min for each simulation with 8 cores. The absolute value of maximun edge size of the tetrahedral mesh used for each simulation was 0.3 ​mm, providing a total of 363586 tetrahedral elements for the native aorta, 405991 tetrahedral elements for the regular repair and 414453 tetrahedral elements for the extended repair geometry. We assume rigid wall with a blood density of 1060 ​kg/m3 and a viscosity of 0.004 ​kg/ms. We simulated 200 time steps (with a step size of 0.005s), and Non-Slip condition at the wall.

The solution was considered converged when residuals dropped below 1x10^−4^. The hemodynamic behavior of the two types of surgery proposed (regular and extended repair) were compared with each other by evaluating the most relevant hemodynamic parameters such as 3D maps of velocity, pressure and WSS [[35], [36], [37]]. The geometry exhibiting lower velocity, pressure drop, and wall shear stress was considered a more hemodynamically efficient solution and selected for 3D printing.

### 2.2 3D print of vascular scaffold

The geometrical shape of the most hemodynamically efficient patch was used for vascular scaffold 3D printing. A hybrid scaffold consisted of a thick grid, printed by FDM which provide support and geometrical shape and a nanofibers’ network using electrospinning to mimic the interstitial architecture. Patches were initially printed as flattened 2D geometries and latter re-shaped to the 3D patient-specific geometry using 3D printed moulds (see supplementary material, Appendix A).

#### 2.2.1 Fused deposition modelling

Macroscopic scaffolds were printed with PCL filament (1.75 ​mm eMorph 3D filament Natural, esun, Shenzen Esun Industrial Co.Ltd) in a FDM 3D printer (Prusa i3 MK3 (Prusa Research a.s., Holešovice, Czech Republic) with a 0.4 ​mm nozzle) with heated bed. This external framework was designed with Prusa Slicer (version 2.1, Prusa Research a.s., Holešovice, Czech Republic) software to provide geometrical shape and support to the subsequent layer of electrospin fibers. Printer head was purged 3 times with PCL filament and cleaned with a wire brush. Printer plate was cleaned with water and 70% ethyl alcohol. The patch was printed at 180 ​°C, 20 ​mm/s speed and 0.1 ​mm layer thickness. The patch was designed using a four-layered grid pattern. The strand thickness was 400 ​μm and distance between strands was 2000 ​μm. The diameter of the patch was 34.8 ​mm. More details of the printing process are provided in Appendix A.

#### 2.2.2 Electrospinning

The first step was the preparation of the solution for electrospinning. The solution consisted of PCL (14% w/w, Mw: 8000, Sigma-Aldrich, US) dissolved in chloroform (Sigma-Aldrich, US)/DMF (N,N-Dymethylformamide, analytical reagent grade, Fisher Chemical, US) (3:2 w/w). All materials were used as purchased, without any further purification. An Electrospinning Professional Lab Device (Doxa Microfluidics, Spain) with 2D injector motion was used to electrospin the PCL solution onto PCL 3D printed scaffolds placed on the metallic collector. We used a coaxial injector to create a shroud of solvent-saturated air stream around the Taylor cone to avoid drying [38,39]. The flow of the solution was fixed by a syringe pump at 0.5 ​ml/h and saturated air by a pressure gauge. We applied positive voltage (3 ​kV) to the injector and negative voltage to the collector (−7 ​kV) to prevent material losses, and the collector to injector distance was 15 ​cm. Detailed electrospinning setup is described in Appendix A, Supplemental Fig. 1. Different masses of PCL nanofibers were deposited in each side of the PCL 3D printed scaffolds to obtain different mesh porosity. Our patches were designed to have two faces: a low-density external face (future adventitia) and a high-density internal face (future intima). The number of nanofibers is determined almost linearly by the time the electrospinning is running, obtaining a weight of nanofibers of 3.1 ​mg in 3 ​min on the high-density internal face of the 3D printed scaffold and of 0.33 ​mg in 20 ​s on the low-density external face. On the other hand, the porosity of the non-woven mat of nanofibers decreases with the running time. Porosity (pore size) was determined from SEM micrographs with the help of ImageJ; at least 20 holes between fibers were measured for each scaffold, and at least 10 scaffolds were analyzed. Lower spray time and density of nanofibers were deposited in the external face to achieve a bigger pore size, as this is where the MSC were seeded to allow gravity deposition and migration. On the other hand, the opposite high-density nanofiber face had two purposes. First, it prevents the MSC from seeping through the patch and more importantly, as it will be the interface with the blood stream, the 10 ​μm pore size will allow the diffusion of nutrients and oxygen into the vascular graft and will potentially allow migration and extension of endothelial cells.

### 2.3 Cell culture

#### 2.3.1 Scaffold preparation

The scaffolds were washed three times in 70% ethanol on both surfaces and later in sterile PBS (Gibco, US) in order to prevent contamination. Finally, the scaffolds were submerged in the sterile cell culture tubes (Sarstedt, Germany), sealed and exposed to ultraviolet (UV) light during 10 ​min for each side.

#### 2.3.2 Mesenchymal stem cell culture and vascular smooth muscle cell differentiation

The MSCs used were Poietics™ human adipose derived stem cells ADSC-PT-5006 (Lonza, Switzerland). The vial was cryopreserved in the first passage (P1) and it contained ≥1,000,000 ​cells. The vial of MSCs was defrosted and then seeded into 5 flasks (2.0 ​× ​10^5^ ​cells) (Thermo Fisher Scientific, US) with 3 ​ml of complete growth medium (Lonza, Switzerland) (Appendix B, Supplemental Table 1). Then the flasks were incubated at 37 ​°C, 5% CO_2_ and 90% humidity. Three to four days after the seeding, MSCs could reach a 90% confluence and were ready for a cell passage. After at least 1 week in culture and between P3 and P6, MSCs were treated with 0.05% trypsin EDTA (Sigma, US) and 2.5 ​× ​10^5^ ​cells were seeded on each patch. To optimize the cell adherence to the scaffold, the 3D patches were inserted in culture tubes (Sarstedt, Germany) with cell medium and 2.5 ​× ​10^5^ ​cells per patch. The lower surface of the patch, with high density of nanofiber was adherent to the tube and the upper suface, with a low density electrospinning and larger pore diameter was in the inner central part of the tube to permit the entrance of the cell in the seeding. The tubes were placed in the incubator on the roller mixer with simultaneous double rotative-oscillating movement and inclination angle of ±4° (RollerMix, Ovan, Spain). After 24 ​h, the patches were removed from the tubes and placed in plates (Thermofisher, USA).

MSCs were differentiated to VSMCs using a differentiation cocktail during 14 days; the medium was gently and completely removed from the tubes every two days. The differentiation cocktail contained the transforming growth factor beta 1 (TGFβ-1) (10 ​ng/ml) (Gibco, US), the platelet-derived growth factor composed by two beta chains (PDGF-BB) (25 ​ng/ml) (Gibco US) and the bone morphogenetic protein (BMP4) (2.5 ​ng/ml) (Promocell, Germany), (Appendix B, Supplemental table 1). [40] Two experimental conditions for each assay were conducted as follow: undifferentiated MSC (MSC) and differentiated MSCs into vascular smooth muscle cells (dVSMC). After 2 weeks in culture, the patches were ready for evaluation.

### 2.4 Evaluation and analysis methodology

#### 2.4.1 Scaffold morphology

Fiber morphology was characterized by field emission scanning electron-focus ion beam microscope (SEM-FIB, Helios Nanolab 650, FEI Europe B.V., Spain). The samples were coated with iridium layer using sputtering (K575, Emitech, US). Fiber diameters were determined from SEM micrographs with help of ImageJ software, measuring of minimum of 50 fibers. To obtain the weight (grams per square centimeter) of the deposited nanofibers, all grids were weighed before and after deposition on a precision weighing (A&D BM-252), each weighing was repeated 3 times.

#### 2.4.2 Graft mechanical properties

In order to characterize the de-cellurarized aortic scaffold mechanically, a custom-made inflation test assembly was implemented to measure the burst pressure $p_{b}$ controlling their deformation rate. The scheme of the inflation test is shown in Appendix C, Supplemental Fig. 2. The assembly consisted of a custom-made pierced chamber filled with water at room temperature (RT) introduced from the bottom and a cover to confine the water. This cover had a hole in the center where a 0.075 ​mm thick latex membrane placed to avoid water leaks and to produce a uniform deformation. Scaffolds were clamped between two constraining plates, making them concentric with the latex membrane (Appendix C, Supplemental Fig. 2 A and B). Water pressure was increased by the controlled injection of 0.531 ​ml/min into the chamber using a Harvard Apparatus 11 Plus 70–2208 Syringe Pump producing implant deformation. Pressure was measured using a high precision manometer. Deformation was indirectly quantified by means of the implant displacement h (Appendix A, Supplemental Fig. 2 D and E) with an optical measurement system mounted over the specimen, which allows a controlled incremental height of 0.1 ​mm (Nikon M Plan 20x/0.40,210/0, Japan). The inflation test consists of measuring the vertical displacement h of a reference point of the implant at the center of its shape when it was submitted to a measured pressure p of the fluid. Thus, deformation ε can be described as

(1) $$\varepsilon =\frac{L-D}{D}=\frac{1}{D}\left(h+\frac{D^{2}}{4h}\right)\arcsin\left(\frac{D}{h+\frac{D^{2}}{4h}}\right)-1$$

where D is the membrane-scaffold contact surface diameter (D=25mm) and L is the length of the arch formed by the deformed implant [41] (Appendix A, Supplemental Fig. 2 B). The number of implants tested was n=15. Maximum stress at burst ($\sigma_{b}$) was calculated according to the law of Laplace for a thin-walled sphere:

(2) $$\sigma_{b}=\frac{p_{b}r}{2t}$$

where t= 0.489 ​± ​0.019 ​mm is the thickness of the scaffold, $p_{b}$ is the burst pressure measured in the inflation tests and *r* is the characteristic radius of the spherical scaffold. It can be demonstrated that:

$$r=\frac{D^{2}+4h^{2}}{8h}$$

The effective Young's modulus *E* at the point of burst can be defined as [42]:

(3) $$E=\frac{\sigma_{b}}{\varepsilon_{max}}\left(1-\nu \right)$$

where $\varepsilon_{max}$ is the strain at failure and ν=0.38 is the Poisson's ratio for PCL [43].

The mechanical properties were compared with previously reported scaffolds’ mechanical properties (See Appendix C, Supplemental table 2) [44].

#### 2.4.3 Assessment of cell viability and infiltration

To assess the cell viability, weekly cell cultures were stained weekly with calcein-AM solution as described by Li.N. et al. [40]. The samples were washed twice with PBS, incubated with calcein in Dulbecco's Modified Eagle's Medium (DMEM) and calcein-AM (10 ​μM)(Sigma, US) during 30 ​min at 37 ​°C, washed twice again and observed under inverted phase-contrast microscope using 4× objective (phase contrast microscope Olympus I×71, Japan). Filter wavelengths of 488 ​nm for excitation and 500–550 ​nm for emission were used to detect hydrolyzed calcein in the cytoplasm. Calcein quantification, expressed in percentage, was obtained using Image J software, calculating the area covered with cells of random squares of each scaffold seeded with MSC snd dVSMC.

Confocal and scanning electronic microscopes were used for evaluation of fiber morphology, diameter, density and spreading coating of the cells over the scaffold surface. First step was an immunofluorescence assay labelling cell structure with *anti*-vimentin and the nucleus with DAPI 1:1000 (Bio-Rad, US) using Alexa Fluor-conjugated secondary antibodies (Appendix D, Supplemental table 3). The images were taken in a confocal inverted microscope scanning Zeiss LSM7 DUO laser with a 20x/0.8 Plan-Apochromat objective. The excitation laser lines for fluorochromes DAPI and Alexa fluor 594 were the 405 ​nm diode and HeNe594, respectively. Second step was the preparation of new samples for scanning electronic microscopy. Samples were fixed with 2.5% glutaraldehyde in 0.1 ​M cacodilate buffer for 2 ​h, then 3 washes were performed in 0.1 ​M cacodilate buffer, a second fixation with 1% osmium tetraoxide was performed for 1 ​h; dehydrated in a gradual concentration of ethanol up to 100%; left to dry and finally coated with a layer of approximately 10 ​nm Au/Pd thickness. The images were acquired on a Zeiss EVOLS15 scanning electron microscope at a voltage of 10 ​kV.

#### 2.4.4 Assessment of cell differentiation

The evaluation of MSC differentiation towards dVSMC was evaluated by Rt-qPCR, western blotting (WB), immunofluorescence, flow cytometry and calcium (Ca^2+^) release analysis.

##### 2.4.4.1 RNA extraction and Rt-qPCR analysis

Before RNA extraction cells were trypsinized from the scaffold and seeded in plates. Then, we began the RNA extraction using “QIAzol lysis reagent” (Qiagen, Germany), similar product to TRIzol. Secondly, to tRNA isolation total RNA was extracted following the kit instructions of miRNAeasy kit (Qiagen, Germany). Reverse Transcription (Rt) was performed by iScript cDNA Synthesis Kit (Biorad, US) (Appendix D, Supplemental table 4). Gene expression quantification was performed using the qPCR (quantitative Polymerase Chain Reaction). Samples were diluted 1:5 in H2O, using triplicates for each condition in a FrameStar 384 Well PCR Plate 384-well plate. Sybr-Green (Qiagen, Germany) reagents and sense and antisense oligonucleotides of each gene were used (Appendix D, Supplemental table 4). The qPCR cycle program was 95 ​°C 20 ​s, 40 cycles at 95 ​°C for 1min and 60 ​°C for 20 ​s in the Viia7 Real-Time PCR System (Applied Biosystems, US).

##### 2.4.4.2 Western blotting

Cells were trypsinized from the scaffolds and they were seeded in plates (Thermo Fisher Scientific, US). Next, protein extraction was carried out with NP40 Cell Lysis Buffer (Thermo Fisher Scientific, US) and quantified by Bradford method. Protein samples were subjected to SDS-PAGE (10% acrylamide) and electrotransferred onto PVDF membranes. After blocking with 5% non-fat dry milk dissolved in Tris-buffered saline containing 0.1% Tween-20 (TTBS) for 1 ​h at 37 ​°C, membranes were probed overnight at 4 ​°C with anti-alpha-smooth muscle actin (α-SMA), anti-smooth muscle protein 22 (SM-22), *anti*-calponin, *anti*-smoothelin and *anti*-GAPDH primary antibodies in TTBS with 1% of bovine serum albumin (BSA) (Sigma, US). After washing, membranes were incubated for 45 ​min RT with a horseradish peroxidase conjugated *anti*-IgG (Cell Signaling, MA, US). Detection was performed in the ChemiDoc™ Touch Imaging System, (Bio-Rad, US). Images were analyzed with Biorad Image Software. (Appendix D, Supplemental table 4).

##### 2.4.4.3 Immunofluorescence staining

Cells were trypsinized from the scaffold and seeded in cover-slips (Thermo Fisher Scientific, US).After being cultured overnight, cells were washed with PBS and fixed with 4% formalin for 20 ​min. Then they were permeabilized with PBS 1X- 0,5% Triton 100X (permeabilization solution) for 20 ​min. Subsequently, the samples blocked with PBS 1X/1% of BSA (Sigma, US)/0.5% Tween 20 (Sigma, US) for 30 ​min.

Then they were incubated with anti-human antibodies including anti- α-SMA, *anti*-SM-22, *anti*-calponin, *anti*-smoothelin, anti-fibroblast specific protein 1 (FSP1) and anti-cluster of differentiation 31 (CD31) during 2 ​h at RT (Appendix D, Supplemental table 3). After being washed, the samples were incubated with Alexa Fluor-conjugated secondary antibodies (Thermo Fisher Scientific, US) at RT for 40 ​min (Appendix D, Supplemental table 3). Then, they were washed and stained with DAPI in PBS 1X to label cell nuclei for 5 ​min. Then they were washed once with PBS 1X and twice with distilled water and finally the cover-slips were mounted on slides with 6 ​μL of DAKO Fluorescence Mounting Medium (Dako, US). The fluorescence images were acquired with direct fluorescence microscopy (Olympus IX-61, Japan) and with confocal microscopy with laser excitation at 405, 458, 476, 488, 496, 514, 561, 594 and 633 ​nm (Leica TCS-SP2-AOBS Spectral Laser Scanning Confocal Microscope, Germany). Images were analyzed with ImageJ software.

##### 2.4.4.4 Flow cytometry

After 14 days of differentiation, MSC and dVSMC were trypsinized from each scaffold with 0.25% for 5 ​min and then transferred into a vial. For cellular antigens analysis, cells were first fixed with cold formaldehyde 4% for 10 ​min at RT. Cells were vortexed intermittently in order to maintain a single cell suspension. The fixation solution was removed through centrifugation at 1500 ​rpm for 5 ​min, and then washed with PBS. Subsequently cells were permeabilized with of 0.1% Triton-X100 (permeabilization solution) for 10 ​min. Then, we added the unconjugated primary antibodies *anti*-SM22, *anti*-calponin, *anti*-smoothelin and *anti*-FSP1 (Appendix D, Supplemental table 3) during 30 ​min at RT. Subsequently cells were washed and incubated with the Alexa Fluor-conjugated secondary antibodies (Thermo Fisher Scientific, US) and the α-SMA Alexa Fluor conjugated antibody (Thermo Fisher Scientific, US) during 20–30 ​min protected from the light (Appendix D, Supplemental table 3). Then cells were washed and resuspended in PBS 1X and analyzed with the flow cytometry. All samples were acquired immediately after the staining protocol on a Canto II flow cytometer (BD Biosciences, US) equipped with three lasers (405 ​nm, 488 ​nm, 640 ​nm). The FACSDiva software 8.0 (BD Biosciences, US) was used for the samples acquisition and analysis.

#### 2.4.5 Functional analysis

MSCand dVSMC were trypsinized from the scaffold and seeded on coverslips to incubate them with DMEM+ 2–5 ​μM Fura-2AM (Thermo Fisher Scientific, US) for 30 ​min at RT, and then cells were washed. For the experiments, a coverslip was placed on the stage of Nikon Eclipse TS-100 inverted microscope equipped with a 20 ​× ​Fluor objective (0.75 NA), as has been previously described 45. Fluorescence images from a large number of loaded single cells were recorded and analyzed with a digital fluorescence imaging system (InCyt Basic Im2, Image Solutions (UK) Ltd., Preston, UK) equipped with a light-sensitive CCD camera (Cooke PixelFly, Applied Scientific Instrumentation, Eugene, US). Changes in the concentration of intracellular calcium [Ca2+]i are represented as the ratio of Fura-2 fluorescence induced at an emission wavelength of 510 ​nm due to excitation at 340 and 380 ​nm (ratio ​= ​F340/F380). Ca2+ influx was calculated as the difference between the peak ratio before and after the addition of drug (Δratio). Calcium leakage was subtracted in every condition. Experiments were performed using a continuous perfusion system in physiological saline solution (PSS) (in mM, pH ​= ​7.4: 140 NaCl, 2.5 CaCl2, 2.7 KCl, 1 MgCl2, 10 HEPES, and 10 Glucose). Drug solution was: endothelin solution (20 ​nM) in PSS [45].

### 2.5 Statistical analysis

Analysis were performed with GraphPad (GraphPad Software, Inc., CA, US). Results are presented as mean and Standard Deviation. The outliers were removed based on results of QuickCalcs, an online tool of Graphpad. We used Shapiro-Wilk as normality test. To compare normal data we used the Ordinary one-way ANOVA or t-student. For non-normal distributed data we used Mann-Whitney and Kurskall-Wallis test. Statistical significant differences were considered when p ​< ​0.05.

## 3 Results

### 3.1 Patch properties

#### 3.1.1 Hemodynamic properties

Physiological neonatal pressure gradients were obtained for each simulation. In the initial native aortic coarctation there was a high velocity blood flow across the coarctation (2.2 ​m/s), a pressure gradient between the ascending and descending aorta of 16 ​mmHg and a high wall shear stress of over 15 ​N/m^2^. The simulation showed that the extended repair resulted in a better surgical repair compared to regular repair. In the regular repair, although pressure gradient dropped to 6 ​mmHg, the blood flow was asymmetrical with a velocity acceleration along the inferior curvature of the aortic arch, resulting in a higher wall shear stress >5 ​N/m^2^ in the repaired distal area. The CFD simulation in the extended repair showed a significantly reduced gradient of only 5 ​mmHg, a more laminar blood flow along the aortic arch and a lower wall shear stress below 5 ​N/m^2^. Based on these results, we discarded the regular repair patch geometry and chose the extended arch repair geometry for fabricating the scaffold.

#### 3.1.2 Mechanical properties

**Fig. 3:**
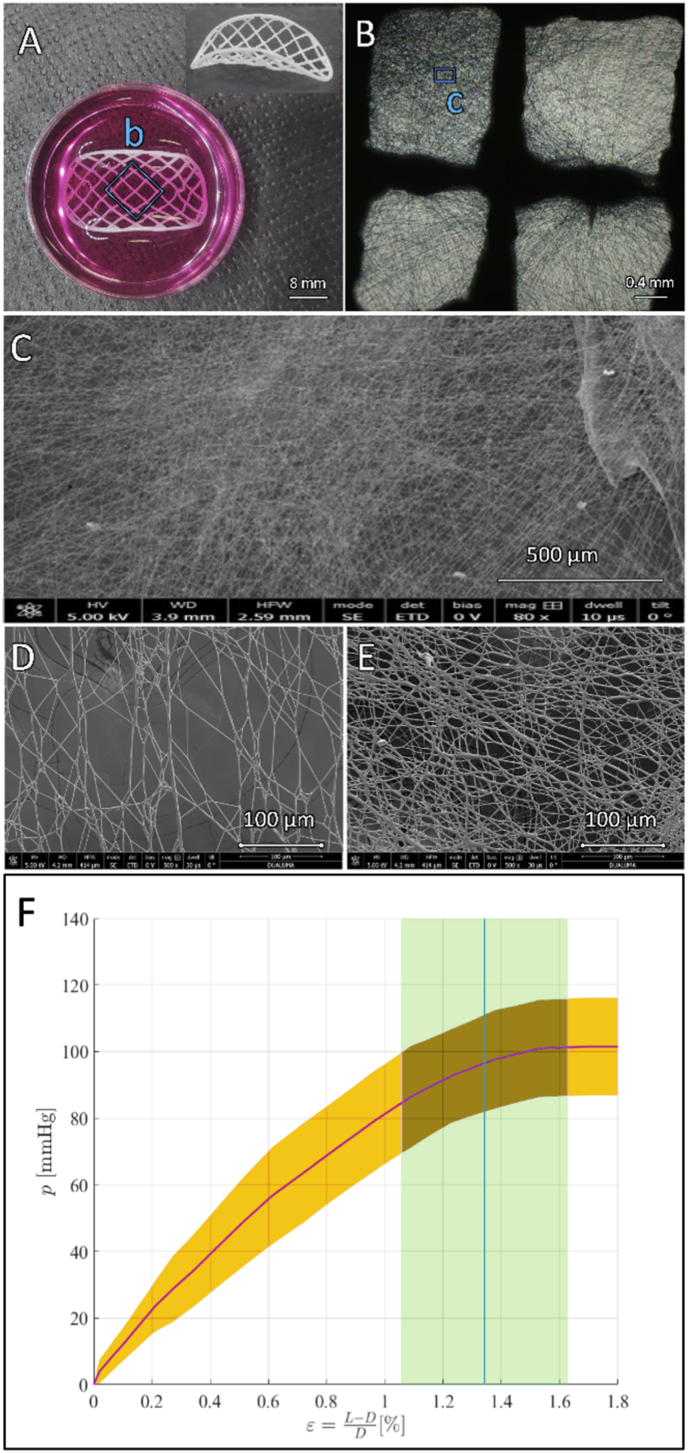
**3D print of vascular scaffold and 3D print of vascular scaffold and pressure-strain implant curve. A.** Macroscopic patch view. The three-dimensional patch geometry and the gross strands printed with fused deposition modelling can be seen. **B.** Microscopic optic view of the grids allows visualization of the nanofibers created with electrospinning. **C.** Electronic microscopy view demonstrating the spatial arrangement and thickness of the nanofibers below 1 ​μm. **D.** Upper surface, low density area demonstrating larger pore diameter. **E.** Lower surface, high density area. **F.** Pressure (p, mmHg), strain (*ε*, %).

Fig. 3 F shows the inflation test pressure-strain curves of the decellularized extended repair scaffold. The pink solid line represents the mean pressure-strain values, and the pink shaded zone represents the pressure standard deviation. The blue solid line represents the mean deformation obtained when burst pressure breaks the implants and blue shaded zones represent standard deviation of the deformation related to burst pressure values. The average burst pressure reported was $p_{b}=$ 101.48 ± 14.7 ​mmHg, which was above the average systolic pressure in neonates and children which ranges between 70 and 100 ​mmHg [46]. Scaffolds presented relatively low deformations until breakage, showing maximum deformation values of $\varepsilon_{max}=$ 1.34 ± 0.29% when burst pressure was accomplished. Using equations (2), (3), results for maximum stress at burst $\sigma_{b}=$ 0.627 ​± ​0.084 ​MPa and Young's modulus E= 29,903 ​± ​7984 ​MPa.

#### 3.1.3 Scaffold porosity

The scaffold porosity and nanofiber density were evaluated by electronic microscopy (Fig. 3 A–E). The upper face (high density mesh, small pore size) collected a mass of 2.11 ​± ​1.2 ​g/m^2^ and an average thickness of 20 ​μm, whereas the lower face (low density mesh, large pore size) collected 0.27 ​± ​0.2 ​g/m^2^ and an average thickness of 7. The distribution of the nanofibers was mildly homogeneous, although nanofibers tend to land preferentially on the center of the FDM grid squares (Fig. 3 B and C). The average nanofibers size distribution was of 350–100 ​nm. Few thicker fibers could be observed, probably due to some isolated process instability. The nanofiber mesh mean porous size was of 80–100 ​μm on the upper face (low-density fibers, Fig. 3 D) and 10 ​μm on the lower face (high-density fibers, Fig. 3 E).

### 3.2 Patch biocompatibility

#### 3.2.1 Cell viability

**Fig. 4:**
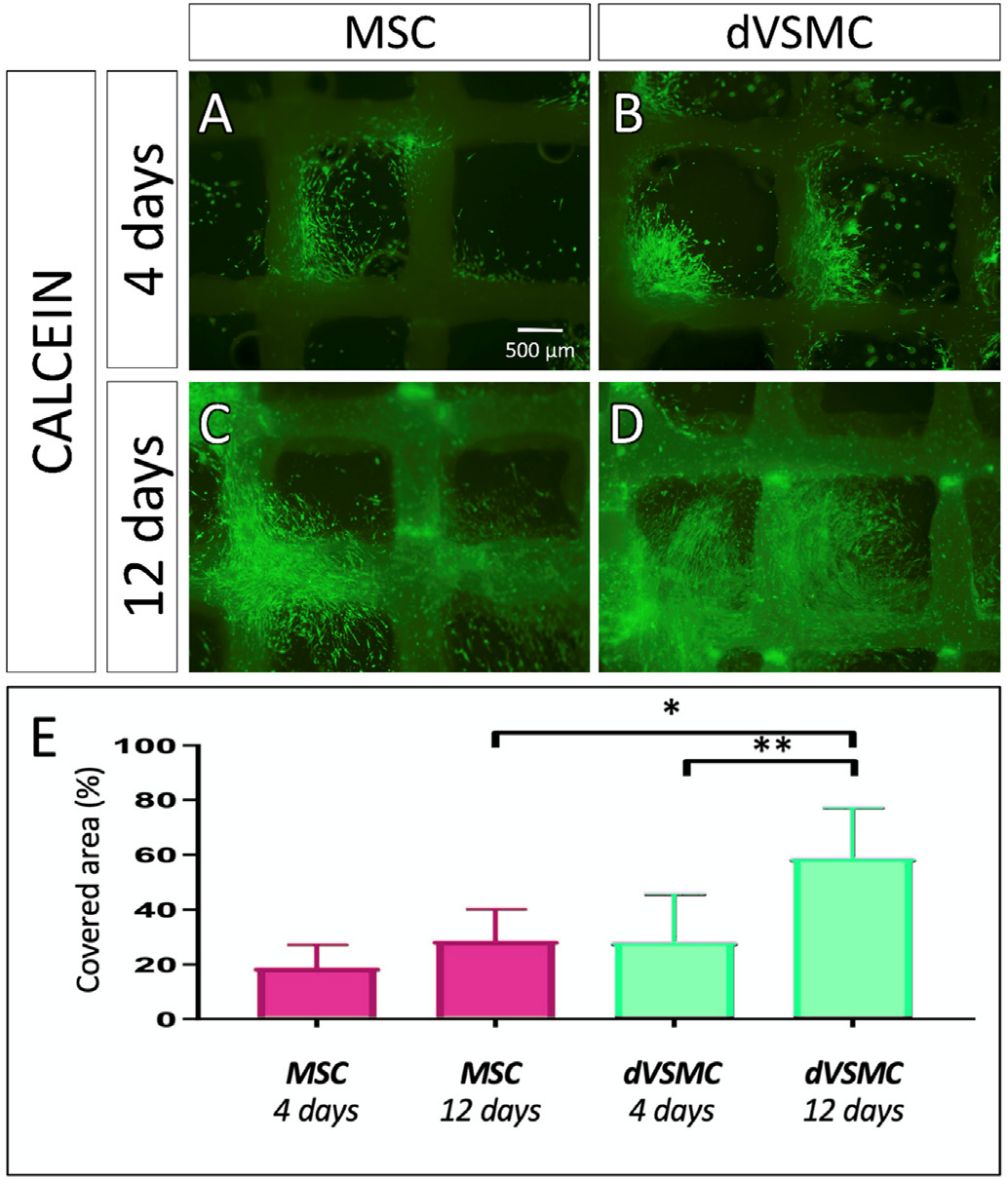
**Assessment of cell viability. A-D.** Viability staining with calcein at day 4 and day 12 in MSC and dVSMC. **E.** Calcein quantification in percentage per unit area in analogous sections in scaffolds seeded with MSC and dVSCM. Data are means ​± ​SD (n ​= ​4–6). “∗” and “∗∗” indicate significance at p ​< ​0.05 and p ​< ​0.01.

**Fig. 5:**
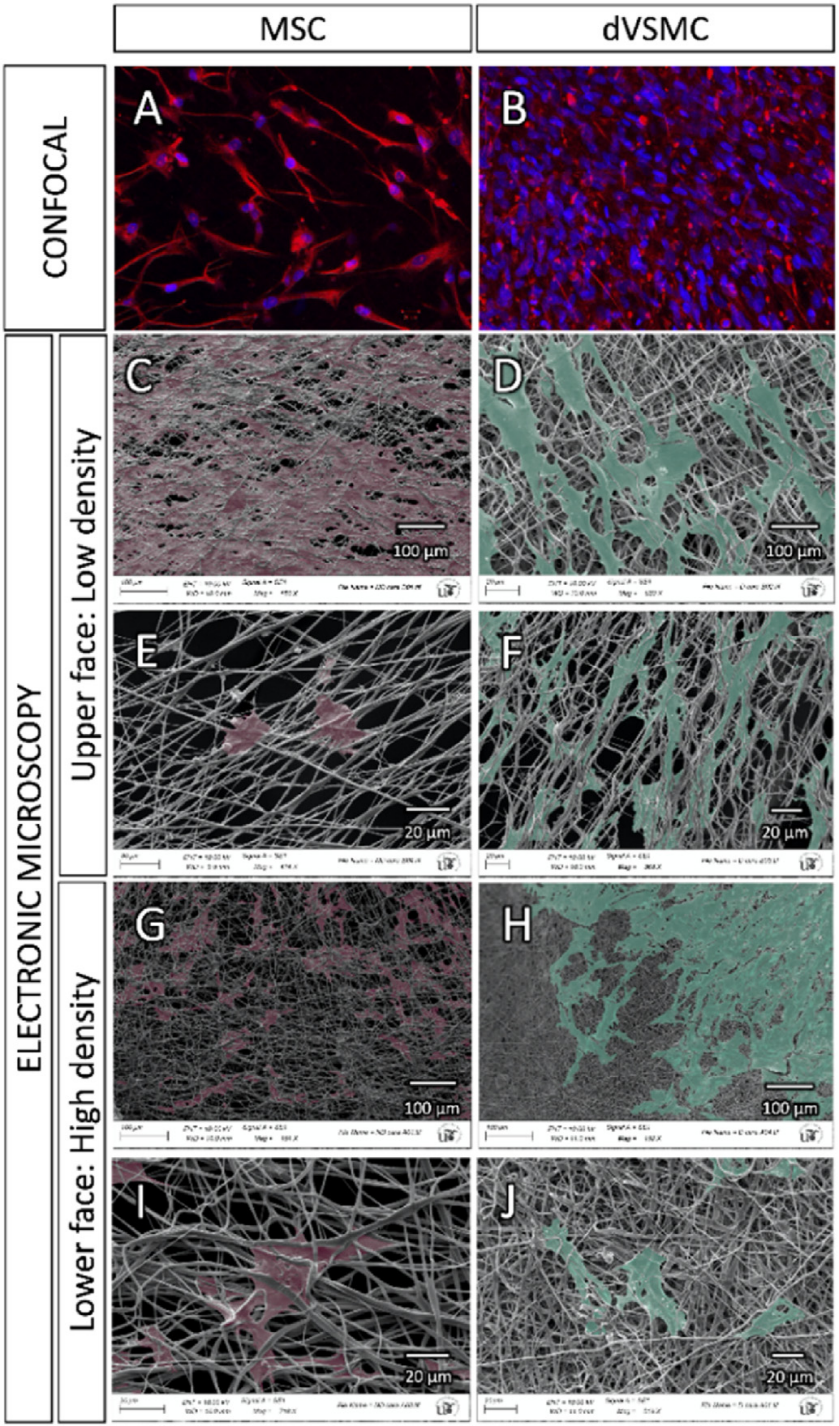
**Penetrance microscopy assays. A-B.** Confocal 3D image of MSC and dVSMC at day 14 in the patch. In red is represented vimentin, cell nuclei are stained in blue with DAPI. In red is represented vimentin, cell nuclei are stained in blue with DAPI. **C-J.** Scanning electron microscopy of both sides of the patch at day 14. **C–F.** Upper surface: Low nanofiber density and larger gaps. This is the graft side where cells are seeded, deposit and gravity-fed through the pores of the patch. **G-J.** Bottom surface: This is the lower side of the graft where cells cannot be strained due to high density of nanofiber and smaller gaps.

Cell viability on patches was evaluated with Annexin V- FITC at day 14th (Appendix D, Supplemental Fig. 4 E) and calcein on the 4th and 12th days after cells seeding, as showed in Fig. 4. Both, MSC and dVSMC patches, showed a good cell viability at day 4, revealing that during the first few days, cells tended to grow and align along the electrospinning fibers. At day 12, dVSMC showed increased cell population compared to MSC (Fig. 5).

At day 14, the scaffolds were labelled with a cytoskeleton marker to study the cellular penetrance through the scaffold (Fig. 5 A and B) and Supplemental Video 2 and Supplemental Video 3. In addition, structural surface analysis was performed with scanning electron microscopy, where both scaffold's surfaces were compared. In the upper face, some cells covered the surface of the scaffold, but most of them seeped through the larger pores (Fig. 5 C–F). The images taken from the lower surface show presence of cells that have migrated to the underside of the scaffold and were trapped by the small pores size (Fig. 5 G–J).

#### 3.2.2 Gene and protein expression of VSMC-specific markers in MSC and dVSMC

**Fig. 6:**
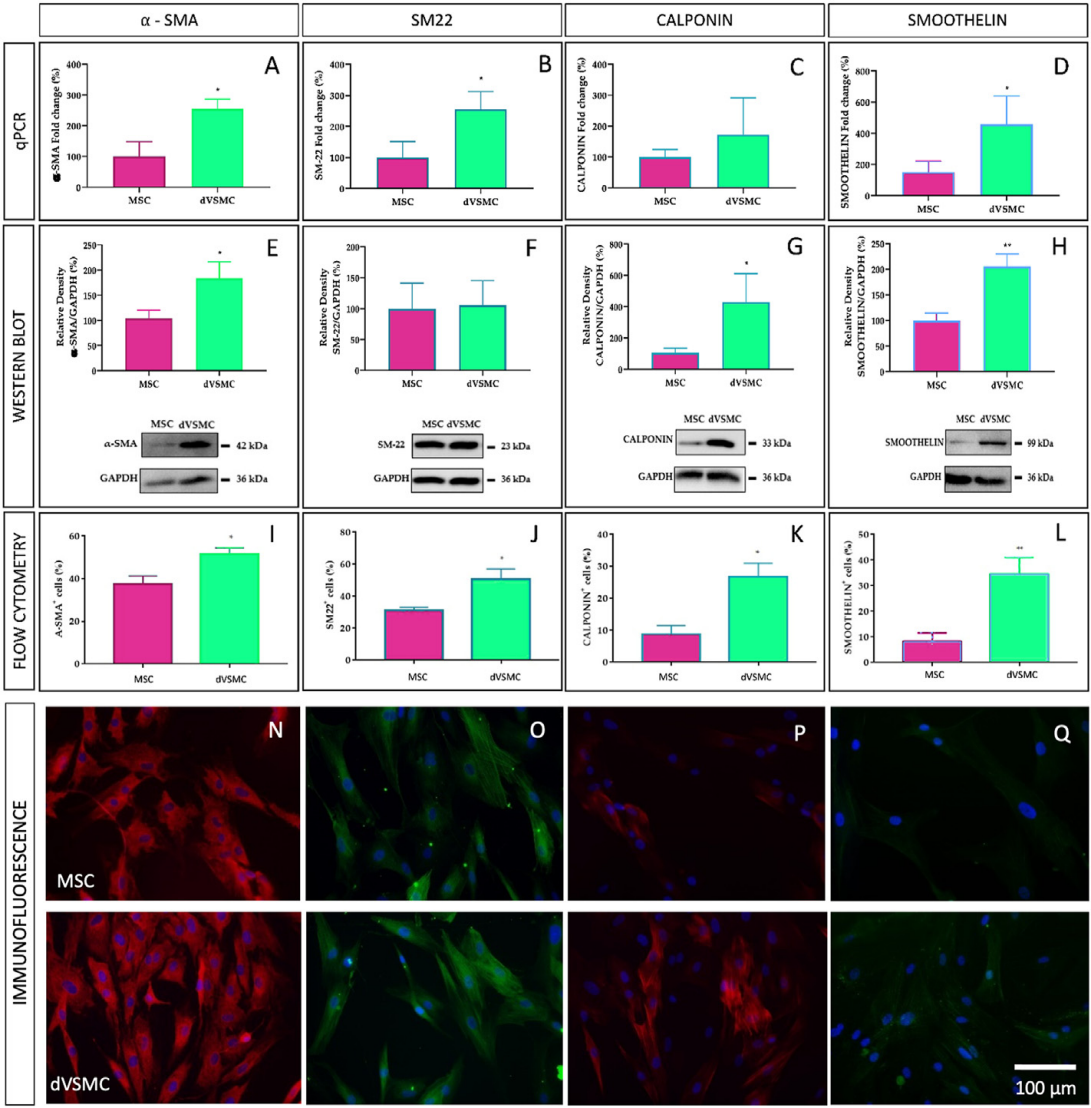
**Differentiation step measure by VSMC-specific marker genes.** Relative mRNA expression, WB plot/bar graph, flow cytometry and immunofluorescence of α-SMA (**A, E, I, N**), SM22 (**B, F, J, O**), calponin (**C, G, K, P**) and smoothelin (**D, H, L, Q**). Results of qRt-PCR for genes expression normalized to endogenous control. Cell nuclei stained with DAPI. MSC: mesenchymal stem cells “Fold change >1” means upregulation, “Fold change <1” means downregulation. Data are means ​± ​SD (n ​= ​6–7). “∗” indicates significance at p ​< ​0.05). MSC: mesenchymal stem cells; dVSMC: differentiated vascular smooth muscle cells.

To study the differentiation of MSC into VSMC, we evaluated genes and proteins expression of various VSMC-specific markers. Firstly, we used Rt-qPCR and WB to assess the expression of α-SMA and SM-22 considered to be expressed at early stages of differentiation, calponin and smoothelin related to intermediate and late stages of differentiation, respectively. Fig. 6 A–D show significant RNA overexpression of VSMC-specific markers in dVSMC compared to MSC.

**Fig. 7:**
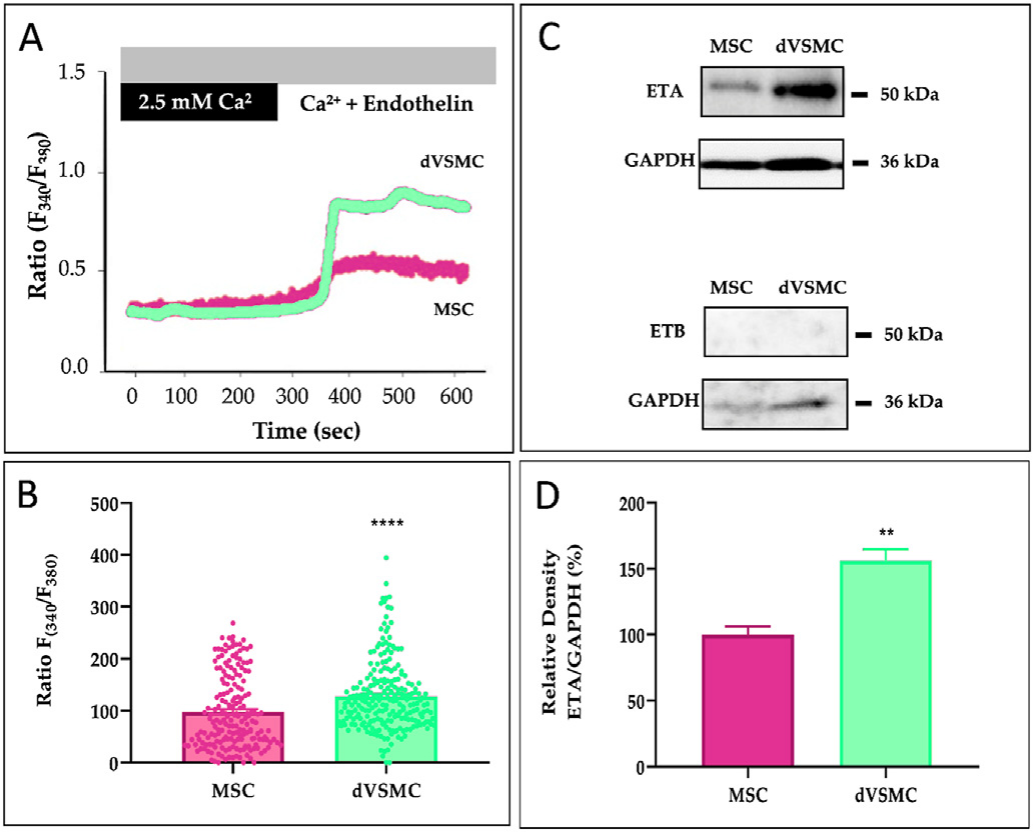
**dVSMC functional Ca**^**2+**^**analysis. A.** Representative traces (left) showing the changes in [Ca^2+]^i in Fura-2 loaded MSC and dVSMC, presented as ratio (F340/F380). **B.** Bar graph illustrating data summary of experiments. PSS solution (2.5 ​mM Ca^2+^) ​+ ​Endothelin (20 ​nM) are the stimulus in [Ca^2+^]i release. **C.** Representative traces showing the changes in [Ca^2+^]i in Fura-2 loaded MSC and dVSMC, presented as ratio (F340/F380). **D.** Bar graph illustrating data summary of experiments. PSS solution (2.5 ​mM Ca^2+^) ​+ ​Endothelin (20 ​nM) are the stimulus in [Ca^2+^]i re-lease. Data are means ​± ​SD (n ​= ​200–250 ​cells). “∗∗” and “∗∗∗∗” indicate significance at p ​< ​0.01 and p ​< ​0.0001.

As shown in WB (Fig. 7 E–H) and flow citometry studies (Fig. 7 I–L), the α-SMA, calponin and smoothelin levels were significantly increased in dVSMC as compared to MSC. Of note, flow cytometry also detected significant overexpression of SM-22 in dVSMC. Moreover, the non-differentiated cells exhibited more fibroblast specific marker 1 (FSP1) than dVSMC (Appendix D, Supplemental Fig. 4 A). Subsequently, dVSMC-specific contractile proteins (α-SMA, SM-22, calponin and smoothelin) accompanied by fibroblast (FSP1) and endothelial (CD31; Appendix D, Supplemental Fig. 4 D) specific markers were immunofluorescence stained. Representative images are shown in Fig. 6 N–Q and summary data in Supplemental Fig. 3 and 4. These results provided robust evidence for the hypothesis that MSC dVSMC from the patch reach high levels of VSMC-specific differentiation markers.

#### 3.2.3 Functional evaluation of the MSC-derived VSMC-like cells

To evaluate whether differentiation of MSC provides functional VSMC, we tested the VSMC-specific response of the intracellular calcium mobilization using a potent vasoactive agonist, the endothelin-1 (ET-1). In experiments performed in isolated dVSMC, the administration of ET-1 (20 ​nm), applied in the continuous presence of extracellular Ca^2+^, evoked a sustained elevation of [Ca^2+^]_*i*_ (Fig. 7 A–B).

Both VSMCs as well as endothelial cells, are used to respond to endothelin. Because of that, the expression of endothelin receptors was examined.

Endothelin Receptor A (ETA) is mainly expressed in VSMCs while Endothelin Receptor B (ETB) is expressed in endothelial cells [47]. Fig. 7 C–D shows that dVSMCs overexpressed ETA as compare to MSC, while no ETB expression was observed. Altogether, these data suggest that dVSMC are functional for vasoconstrictor stimulus involving Ca2+ entry.

## 4 Discussion

The ideal patch should fulfill the following requisites: hemodynamic (optimal shape to reduce resistance to blood flow), resistant (ability to elongate by damping the arterial pulse wave without tearing), material biocompatibility (the ability to be integrated in the surrounding tissue with an appropriate host response), bioabsorbable (capable of being absorbed and replaced by living tissue), growth (adaptation to patient's growth) and regeneration (ability to self-repair preventing thrombosis and stenosis).

### 4.1 Hemodynamic properties

Surgical patch reconstruction is a fundamental technique in a wide variety of diseases. It largely depends on the surgeon's craftsman expertise to intraoperatively cut and model a patch, which fits to the existing defect. Although most of the times the repair may seem acceptable, surgical correction is performed under cardiac arrest in a bloodless aorta. After flow restoration, small anatomical variations are known to have hemodynamic alterations and thus impact on cardiac function [48]. Itatani et al. demonstrated the impact of smooth aortic arch reconstruction in altered wall shear stress and energy loss [3]. These non-laminar flows and altered wall-shear stress affect the endothelial homeostasis, which in turn causes late vascular remodeling, atherosclerosis, re-stenosis and aneurysm [49,50]. The use of computational fluid dynamics to evaluate mechanical performance indices have been already used to help predicting the best patch design customized to patient-specific anatomy and achieve the ideal surgical result [10,11,51]. In our case, although both surgical repair geometries looked almost identical (see Fig. 1), hemodynamic flow parameters were completely different (Fig. 2). From all hemodynamic parameters that can be used to describe complex flow dynamics after aortic surgical repair [52], such as velocity, pressure, oscillatory shear index, helicity, energy loss and eccentricity; WSS nominates as one of the best variables to predict atherosclerotic plaque formation and poor outcomes [53,54]. Therefore, we focused on wall shear stress when comparing the two proposed surgical repair techniques. The extended repair exhibited the lowest WSS and pressure gradient, which makes it more favorable surgical solution and was selected for 3D printing.

We acknowledge that our CFD methodology is a fair non-invasive to estimate hemodynamic conditions, however it has several limitations. For example, we used rigid wall boundary conditions and a steady-state flow rather than full fluid solid interaction and pulsatile flow, which is more physiological. As indicated in previously published material, in the context of certain clinical questions, the simpler analysis methods may be a cost-efficient solution [55], which may capture the important characteristics of the flow field [34,35]. We obtained physiological values for pressure gradient, which normally in neonates and children ranges between 70 and 100 ​mmHg for systolic blood pressure and 50–70 for diastolic blood pressure. In future contribution we want to improve the use of rigid walls using FSI (Fluid Structure Interaction) simulations for the pulsatile flow regime, adding mechanical properties of the patch in the simulation as described by Figueroa et al. [56], and adding mechanical properties of the vessel and the scars produced by the surgery.

### 4.2 Mechanical properties

We successfully implemented inflation tests for mechanical characterization of the 3D printed de-cellularized vascular scaffolds, demonstrating that they showed adequate functionality for pressure values under p_b_ ​= ​101.48 ​± ​14.7 ​mmHg, which implies a maximum stress at burst σ_b_ ​= ​0.187 ​± ​0.027 ​MPa. These mechanical properties were compared with other vascular scaffolds found in the literature [44]. Scaffolds such us collagen gel based, present values of burst pressure p_b_ ​= ​71 ​± ​4 ​mmHg and maximum stress σ_b_ ​= ​0.058 ​MPa, which are lower than the results for the implants tested in this work. On the other hand, other authors reported that decellularized tissue scaffolds and human internal mammary artery present higher values for these mechanical properties [44]. Therefore, it should be noted that implants tested in the present work could be used in applications where low burst pressure conditions are required such as for surgical repair of veins, pulmonary arteries and systemic arteries in children [57]. However, the mechanical properties achieved may not be adequate for hypertensive adult population which easily exceeds 140–180 ​mmHg. Regarding deformations, implants considered in this study break when strain reaches values of *ε* ​= ​1.34 ​± ​0.29%. This strain value shows that this scaffolds present low deformations in the working pressure range before break. The Young's modulus obtained for the implants was E ​= ​8.99 ​± ​1.93 ​MPa, which represents an intermediate value when compared to results in the literature [44]. In future pre-clinical animal studies, we will evaluate the mechanical properties of cellularized patches to characterize the changes as cells will potentially replace polycarprolactone with native interstitial fibers of collagen and elastin.

### 4.3 Hybrid 3D printing

The application of a hybrid approach combining FDM and electrospinning to fabricate a biopolymeric scaffold for vascular tissue engineering has been previously published [58]. FDM enhances resistance providing better mechanical properties while electrospin fibers mimics ECM structure and topology providing an improved surface to cell attachment and proliferation [59]. Compared to previous studies where generic tubular scaffolds were fabricated [58], we implemented the fabrication process by printing patient-specific geometry to facilitate surgery. Our method is reproducible and robust but there is a large margin for implementation. The next line of research will be focused on fabricating more complex geometries and incorporating additional agents to PCL solution such as antibiotics to avoid development of bacterial infections such as endocarditis [60], heparin to prevent thrombosis [61] and growth factors to improve cellular growth and differentiation [23]. One of the limitations of our study is that we did not fabricate more complex hyperbolic paraboloid shapes using FDM. The electrospinning process also has several limitations. It does not distribute the fibers homogenously along the surface and also the deposit of fibers inside of convex and hollow scaffolds is a challenge. As a future line of development, we are working on the creation of aortic segments by printing the polyhedral net of the vessel, projecting the fibers on the surfaces and closing the piece to achieve the customized 3D structure. Future work will also consider using a mixed solution of polycaprolactone, collagen and elastin in order to avoid problems associated with compliance mismatch”

### 4.4 Pore size

Our strategy included a dual gradient approach for electrospinning as matrix pore size is key in cell migration, proliferation, and differentiation [62,63]. The upper (external) surface of the scaffold (Graphical Abstract and Fig. 3 D) required a low density of PCL fibers. Cell migration through the scaffold require larger pore size above 3–12 ​μm, but it largely depends on the transplanted cells. Endothelial cells are able to migrate through pore size of 10–25 ​μm, fibroblasts 20–100 ​μm and MSC 50–200 ​μm [[23], [64], [65]]. We achieved an average pore size around 80 ​μm in the upper face to allow MSC migration by gravity and depositing inside the patch. On the other hand, the lower face surface required a denser mesh of nanofibers for two main reasons. One is to prevent the seeded MSC from migrating, breaking through and out of the patch by gravity. However, the lower face of the patch should not be impermeable or completely covered by fibers as the second requirement is that it should allow diffusion of oxygen and nutrients. The lower surface of the patch will be in contact with the blood flow stream during the future in-vivo experiments. The average pore size in the lower (internal) surface of the scaffold (Graphical Abstract and Fig. 3 E) was 10 ​μm to act as an interface with the blood stream preventing cells from leaking out by gravity during cell culture, but allowing nutrients to diffuse into the patch after the in-vivo grafting. The cell infiltration was demonstrated using confocal microscopy (See Supplemental Video 2,3) and inferred from the electronic microscopy images of the lower face of the patch (Fig. 5 I and J). Cells were enclosed behind the PCL fibers and due to the small pore size of 5 ​μm penetration through this surface is not possible. Our hypothesis is that this suggests that there was a cell migration of cells deposited on the upper surface of the patch through the thickness of the scaffold. We acknowledge that future investigations should include further experimental data on porosity and degree of cell infiltration.

### 4.5 Patch biocompatibility

Biodegradable polymers and polyesters like polyurethanes, poly-lactic acid and PCL have good biocompatibility properties allowing proliferation of endothelial cells onto the luminal side of the graft [66]. We have demonstrated that PCL is a non-toxic polymer, does not affect viability of the MSC nor dVSMC, at least in the short term. Future studies with longer follow-up should evaluate the rate of biodegradability of PCL and also the integration with the host tissue, the inmune-mediated reaction and inflammation [24,67]. As a limitation of the study, we did not evaluate the potential for compliance mis-match between the patch and the native tissue.

### 4.6 Cell differenciation

Transplantation of dVSMCs from pluripotent or multipotent stem cells is a promising cellular therapy to new cardiovascular diseases approach due to the limited source of VSMCs [26,68]. However, the differentiation toward a functional and clinically compatible vascular cells to generate TEVGs remains a major challenge. In this study we have assessed the viability, infiltration, recovering and differentiation of MSCs to dVSMCs in personalized 3D scaffolds.

Many studies described the importance of the cell-cell [69] and cell-matrix [70] interactions and the addition of combining growth factors in the proliferation and differentiation of MSC towards dVSMCs [71]. On the one hand, co-culture of VSMCs with endothelial cells appears to boost the cell proliferation [72]. Few studies described the combination of fibroblast and extracellular matrix (ECM) as enhancer of cell growth and differentiation toward VSMC [40]. Although we achieved differentiation of MSC towards functional VSMC-like cells using a simple but effective cocktail of differentiation factors, in future studies we will explore the co-culture with endothelial cells. As ECM is known to facilitate the cell growth, natural decellularized ECM scaffolds are extensively used with many applications in regenerative medicine and tissue engineering, besides migration and cell adhesion assay [73].

Since the availability of VSMCs and natural ECM scaffold to support cell growth is particularly poor, especially in the pediatric patients, in recent years the research pushed for alternative solutions with novel biocompatible polymers using the electrospinning, the decellularization, the lyophilization, the 3D printing [74] and the 2D and 3D-bioprinting techniques [75]. 3D printing is still not so implemented in the development of vascular grafts because of its challenging application in small structures. Nevertheless, our vascular patch includes the application of multiple techniques of tissue engineering. It combines a 3D printed scaffold of PCL, which exhibits elasticity, a double electrospinning with a different porosity, allowing the cells seed and penetration in it from one side but not their leakage from the other, and the cell differentiation towards dVSMC.

It is well known that a combination of multiple differentiation factors exerted synergistic effect on the MSC differentiation [76]. Here we used TGFβ1, PDGF-BB, and BMP4 for inducing the differentiation of MSC into dVSMC. Other authors bet using vasoactive peptides [[77], [78], [79]], pleitropic lysophospholipids [80], heparin [81] and so on.

Our results reveal that this cocktail of differentiation factors was effective in successfully differentiating MSC toward dVSMC phenotype expressing VSMCs-specific markers (α-actin, SM-22, calponin and smoothelin). In addition, dVSMCs were functional since their stimulation with ET-1 increased efficiently the [Ca^2+^]i. We also detected an overexpression of ETA considered specifically expressed in VSMC than in endothelial cells [47]. Interestingly, both MSCs and dVSMCs showed a Ca^2+^ response to ET-1, whereas these responses were exacerbated in dVSMC, which correlates with the strong expression of ETA in those differentiated cells. Herein, we measured Ca^2+^ response stimulating MSC and dVSMCs with ET-1, meanwhile other authors evaluated functional responses with vasopressin, oxytocin, noradrenaline, serotonin, angiotensin-II or carbachol [82,83].

Taken together, these findings did not only underscore that VSMCs are able to grow, expand and infiltrate in the 3D personalized scaffolds, but also that they expresse contractile capacitative proteins of VSMCs and functional agonist response. These results provide faith to a new fate for engineered vascular tissues.

The greatest advantage of our bioabsorbable scaffold is that in the coming years we could have for each patient a customized and personalized patch with autologous mesenchymal cells differentiated in dVSMC, avoiding immunological responses or a future replacement surgery procedure.

## 5 Conclusions

Great advances have been made in the development of 3D printed tissue engineered grafts over the recent years [12]. To the best of our knowledge, this is the first study to comprehensively combine previously published strategies for the obtention of personalized TEVG. These strategies range from patient-specific anatomical design, computation fluid dynamic validation, fabrication of the scaffolds, assessment of mechanical properties and including MSCs culture and differentiation towards dVSMC. Fused deposition modelling allowed the creation of customized geometry while providing strong mechanical properties. Electrospin nanofibers provided a biocompatible matrix for cellular attachment, proliferation and differentiation. By controlling fiber diameter and deposited density, we optimized porosity to allow cell migration through the entire thickness of the scaffold. However, one of the main limitations of this in-vitro study is that it requieres pre-clinicial animal studies for validation of scaffold degradation, long-term mechanical properties and immune host compatibility. Understanding the VSMC plasticity and molecular mechanisms controlling maturation, repair and regeneration would be key for the future of TEVG. Newborns and children with CHD may be the ideal candidates for the potential clinical translation of this research. After diagnosis, MRI or CT imaging could be used for designing patient-specific vascular graft. In the meantime, multipotent MSCs extracted from subcutaneous fat puncture may be used for graft harvesting and differentiation towards VSMCs. Then the vascular graft based on autologous patient's cells and specifically designed geometry could be used to repair the aortic arch with the potential of adequate blood flow hemodynamics, adequate growth and improved long-term outcomes.

## Author contributions

Conceptualization, I.V. and A.O.; methodology, I.M., E.B., G.G., J.S., A.D., P.P-A. and P.G-P.; software, G.G. and J.S.; validation, I.G-L., E.R., Y.S., K.H., and T.S.; formal analysis, I.M., E.B., G.G., J.S., A.D., P.P-A. and P.G-P.; investigation, I.V., I.M., E.B. and I.G.L.; resources, I.V., A.O. and T.S.; data curation, I.V.; writing—original draft preparation, I.V., I.M., E.B., G.G.; writing—review and editing, I.G-L., E.R., J.S., K.H., A.M., T.S. and A.O.; visualization, I.V., I.M. and E.B.; supervision, I.G-L., E.R., K.H., and T.S.; project administration, I.V.; funding acquisition, I.V.. All authors have read and agreed to the published version of the manuscript.

## Funding

This research was funded by ​Instituto de Salud Carlos III ​through the projects ​PI17/01409, PT20/00069 (Plataforma ISCIII de Biobancos y Biomodelos) and ​PI20/00467 ​(Co-funded by European Regional Development Fund/European Social Fund ​“A way to make Europe"/"Investing in your future”) ​and by the Foundation ​‘Menudos Corazones to help children with heart disease' and the Spanish Society of Paediatric Cardiology and Congenital Heart Disease (SECPCC), grant number ​‘Menudos Corazones 2020’. This work was also supported by the Spanish Ministry of Economy and Competitiveness (PID2019-104084GB-C22) and ANID – Millennium Science Initiative Program – ICN2021_004 and ANID – Millennium Science Initiative Program – NCN17_129, ANID FONDECYT de Iniciación en Investigación #11200481, ANID FONDECYT #1181057.

## Institutional review board statement

The study was conducted according to the guidelines of the Declaration of Helsinki, and approved by the Ethics Committee for Biomedical Research ethics in Andalucia, Hospital Virgen del Rocio, Seville, Spain (1561-N-17, 3D bioprint, March 2018).

## Informed consent statement

Written informed consent has been obtained from the patient's caregivers to publish the clinical images in this paper.

## Declaration of competing interest

The authors declare that they have no known competing financial interests or personal relationships that could have appeared to influence the work reported in this paper.
