# Supplementary material for: Tissue engineered in-vitro vascular patch fabrication using hybrid 3D printing and electrospinning
Source: Mater Today Bio. 2022 Apr 14;14:100252. doi: 10.1016/j.mtbio.2022.100252 (PMC9059085; doi:10.1016/j.mtbio.2022.100252)
Supplement: Multimedia component 4 [file mmc4.docx]

**Supplementary Data**

**Appendix A: 3D printing**

**Patch design and fused deposition modelling**

Several macroscopic patch designs were printed and tested. Sinusoidal and grid patterns with different densities were printed. Sinusoidal pattern was discarded because the patch was not strong enough and many pieces were deformed and broken during the extraction of the printer bed. Grid was the selected pattern because it is stronger, and the patches were easier to manipulate and extract from the printer plate (Figure A1).


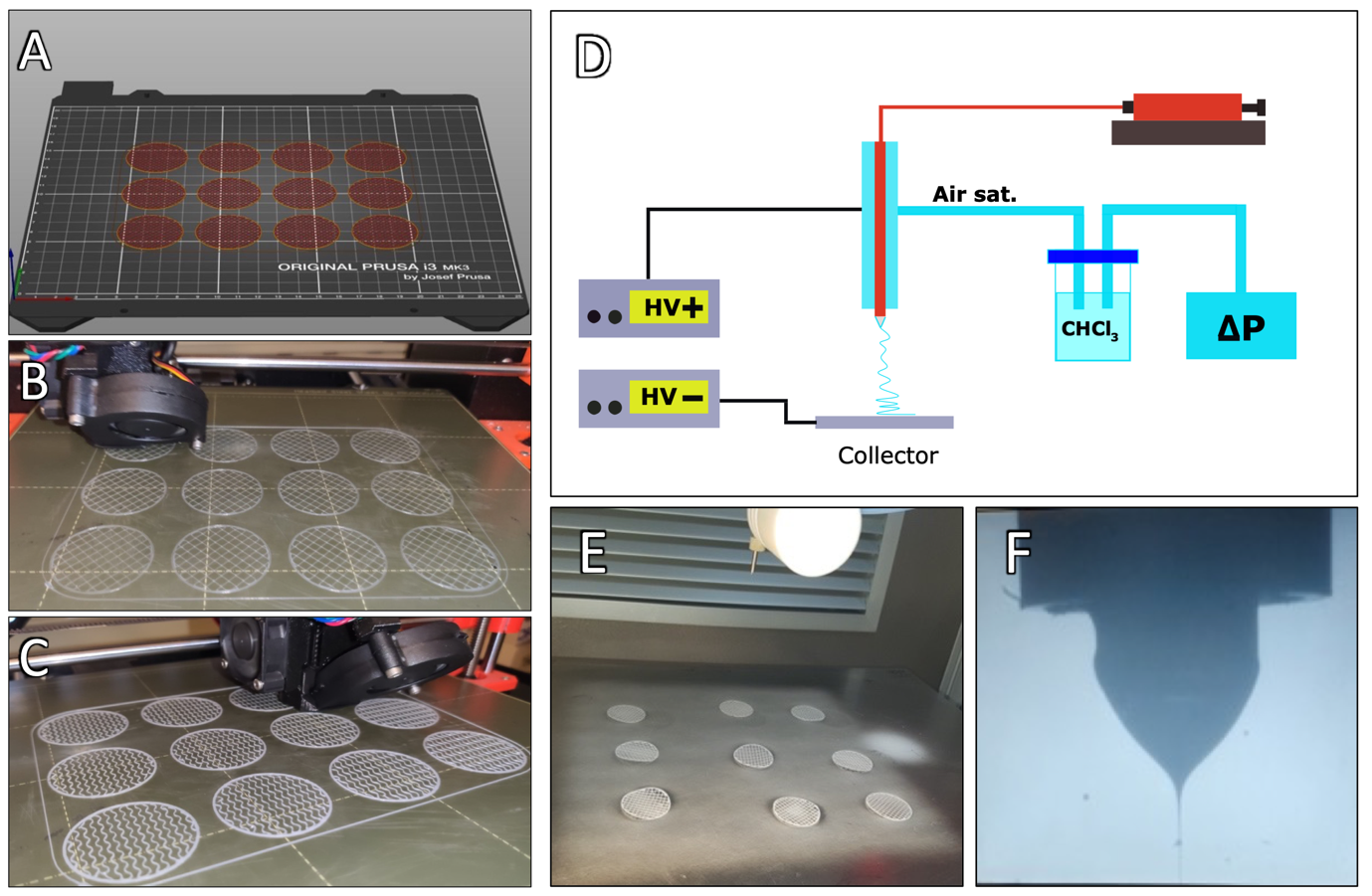


**Figure 1. Patch FDM geometrical design and electrospinning technique. A**. 12 circular patches calculated in PrusaSlicer software previous to printing. **B**. Grid geometrical 3D print. **C**. Sinusoidal geometrical 3D print. **D-F**. Electrospinning technique.

The nozzle used in the 3D printer was a 0.4 mm this is the reason why the macroscopic lines in the printed patches have 0.4 mm wide lines. The separation between lines of 2 mm was selected in order to allow the fibers of the posterior electrospinning process to cover the space between the macroscopic structures. Higher space may result in problems with the coverage of the scaffold and smaller space in clogging of the gaps.

The geometry was processed in PrusaSlicer 2.1.0 software (Prusa Research, Holešovice, Czech Republic) and sent to the 3D printer Prusa i3 MK3 (Prusa Research, Holešovice, Czech Republic). All patches were fabricated in eMorph PCL filament (Esun 3d filament, Shenzhen, China)) by FDM modelling.

Printer head was purged 3 times with PCL filament and cleaned with a wire brush. Printer plate was cleaned with water and 70% ethyl alcohol. PCL filament is difficult to print so many tests were performed before the first successful prints. The material is sticky and it melt at a low temperature. After many tests and experiments the printing parameters selected were: nozzle temperature 180 °C, bed temperature 40°C, 20 mm/s speed and 0.1 mm layer thickness.

The patch was designed using a four-layered grid pattern. The strand thickness was 400 μm and distance between strands was 2000 μm. The diameter of the patch was 34.8 mm.

Once the patches were printed using FDM technology, the electrospinning process was used.The number of nanofibers is determined almost linearly by the time the electrospinning is running, obtaining a weight of nanofibers of 3,1 mg in 3 minutes on the high-density internal face of the 3D printed scaffold and of 0,33 mg in 20 seconds on the low-density external face.

**Appendix C: Graft mechanical properties**

**
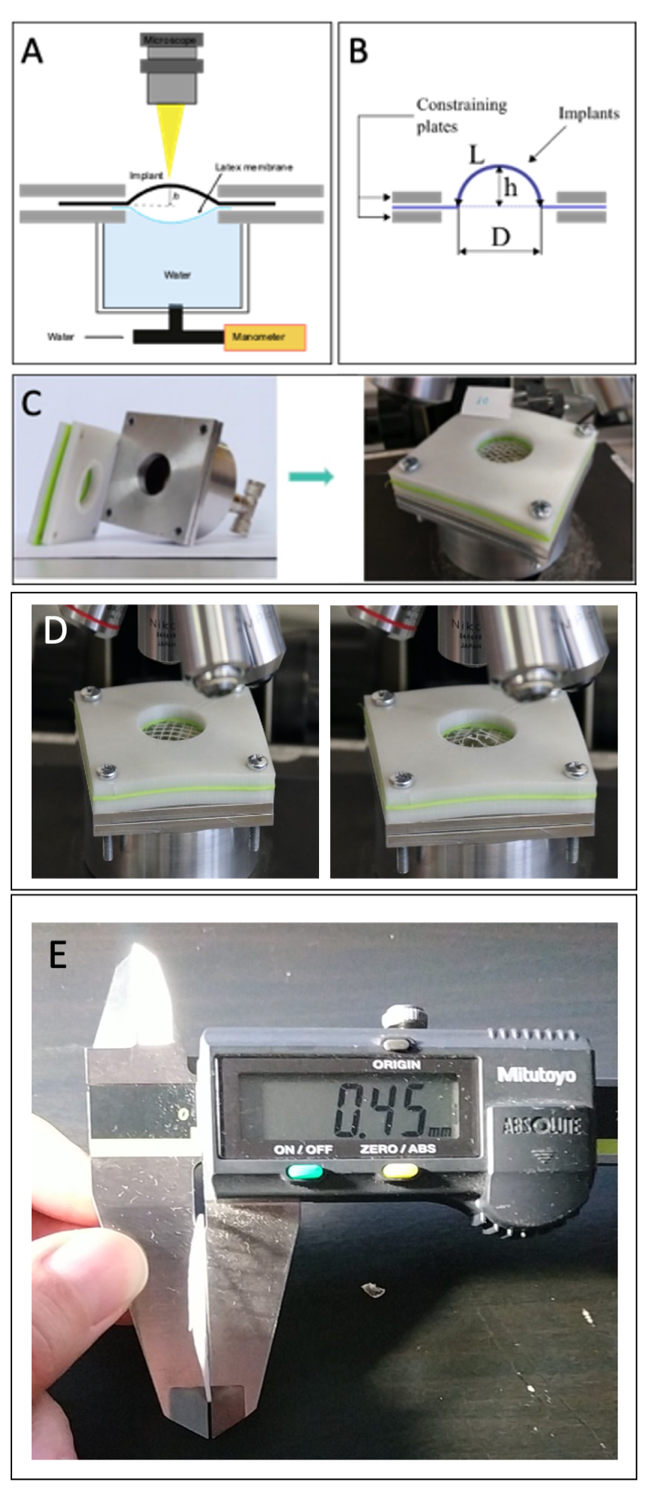
**

**Figure 2. Custom-made inflation test device. A**. Inflation test scheme; **B**. diagram of the aortic implants under pressure; **C**. experimental assembly; **D.** Deformation of the scaffold, a) prior to bursting and b) after bursting; **E.** Scaffold thickness measurement using a high precision digital caliper.

**Appendix D: Evaluation methodologies**


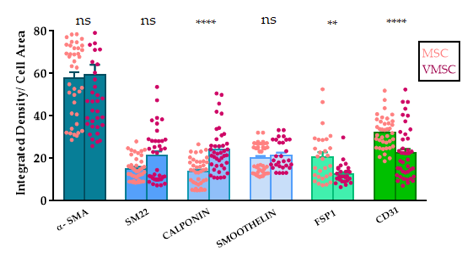


**Figure 3. Immunofluorescence quantification by ImageJ.** Data are means ± SD (n =4). “**”, “****” indicate significance at p< 0.01 and p<0.0001.


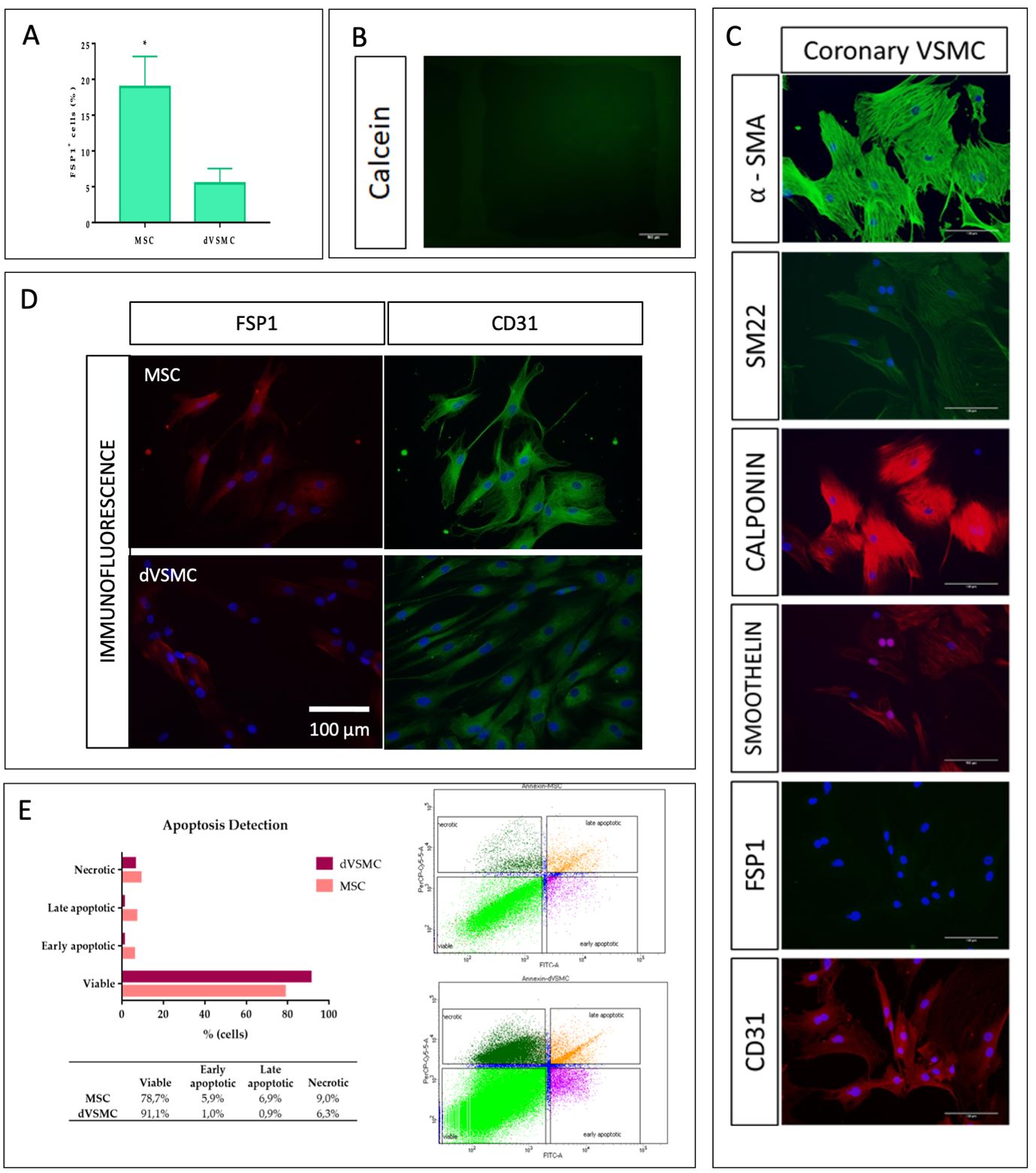


**Figure 4. Panel of flow cytometry and immunofluorescence controls. A.** FSP1 expression measured by flow cytometry. Data are means ± SD (n =4). “*” indicates significance at p< 0.05; **B.** Calcein negative control, performed in a scaffold with no cell seeded. **C.** Immunofluorescence staining. VSMC Obtained from coronary artery labeled with VSMC-specific proteins (α-SMA, SM22, calponin, smoothelin) as well as fibroblast (FSP1) and endothelial (CD31). Cell nuclei stained with DAPI. VSMC: differentiated vascular smooth muscle cells. **D.** Immunofluorescence staining of FSP1 and CD31; **E.** Cell viability with Annexin V- FITC staining. Stained live cells were stained with Annexin V-FITC staining following the flow cytometry protocol (TACS Annexin V kits 4830-250-K, Trevigen, USA). To obtain cells for the flow cytometry assay, the scaffolds were initially trypsinized to obtain MSC and dVSMC cultured for 14 days. Then the staining protocol was performed as following the manufacturer instructions. These results show that in both conditions cells were mostly viable, with a low rate of early and late apoptosis. Necrosis was less than 10%, but we think it is probably due to the trypsinization, which was performed to remove the cells from the scaffolds.
