# Supplementary material for: Tissue engineered in-vitro vascular patch fabrication using hybrid 3D printing and electrospinning
Source: Mater Today Bio. 2022 Apr 14;14:100252. doi: 10.1016/j.mtbio.2022.100252 (PMC9059085; doi:10.1016/j.mtbio.2022.100252)
Supplement: Multimedia component 5 [file mmc5.docx]

**Supplementary Data**

**Appendix B: Cell culture**

In order to prepare the complete Growth Medium for MSC, the contents of the MSCGMTM SingleQuots ^TM^ Supplements and Growth Factors (Lonza, PT-4105) was added to the 440 ml of MSCBMTM Mesenchymal Stem Cell Basal Medium (Lonza, PT-3238); then it was aliquoted and stored at +4°C. The medium, enriched or not with the GFC, was replaced every two days.

**Table 1.** Complete Growth Medium Preparation and mesenchymal cell differentiation cocktail.

| **Name** | **Composition** | | | **Reference** |
| --- | --- | --- | --- | --- |
| MSCBMTM Mesenchymal Stem Cell Basal Medium | 440 ml of adipose-derived stem cell basal medium | | | (Lonza, PT-3238) |
| MSCGMTM SingleQuotsTM Supplements and Growth Factors | 50 ml of fetal bovine serum (FBS)  5 ml of L-glutamine  0.5 ml of gentamicin-amphotericin B (GA-1000) | | | (Lonza, PT-4105) |
|  | **Concentration to resuspend the lyophilized factor** | **Required concentration for the differentiation** | **Required volume for the differentiation per 1 ml of medium** |  |
| 10 µg of TGF-β1 | 0.1 mg/ml in H2O with 0.1% of bovine serum albumin (BSA, A2153, SIGMA) | 10 ng/ml | 0.1 µl | Thermofisher, PHG9202 |
| 50 µg of PDGF-BB | 0.1 mg/ml in 100 mM of acetic acid with 0.1% of bovine serum albumin (BSA, A2153, SIGMA) | 25 ng/ml | 0.25 µl | Thermofisher, PHG0046 |
| 10 µg of BMP4 | In 4Mm of HCl with 0.1% of bovine serum albumin (BSA, A2153, SIGMA)) | 2.5 ng/ml | 0.1 µl | Promocell,  C-67313 |

**Appendix C: Graft mechanical properties**

**Table 2.** Comparison of mechanical properties of scaffolds [29]

| **Scaffold** | **Burst Pressure**  **(mmHg)** | **Maximum stress**  **(MPa)** | **Elastic Modulus**  **(MPa)** |
| --- | --- | --- | --- |
| 3D-printed implants (present work) | 101.48 $\pm$14.7 | 0.187 $\pm$ 0.027 | 8.99 $\pm$1.93 |
| Human internal mammary artery | 3196 $\pm$1264 | 0.443 $\pm$0.055 | 45.1 $\pm$ 16.8 |
| Collagen gel based | 71$\pm$4 | 0.058 | 0.142 |
| Cell sheet engineering | 3490 $\pm$ 892 | >3 | >20 |
| Decellularized tissue scaffolds | 2400 | 1.618 | 7.41 |

**Appendix D: Evaluation methodologies**

**Table 3.** Primary and secondary antibodies.

| **Name** | **Immunofluorescence Working Concentration** | **Flow Cytometry Working Concentration** | **Western blot Working Concentration** | **Reference** |
| --- | --- | --- | --- | --- |
| **Primary antibodies** | | | | |
| Anti-α-actin | 1:400 | - | 1:500 | Sigma, Red A5228 |
| Anti-calponin | 1:500 | 1:100 | 1:1000 | Genetex, GTX64283 |
| Anti-smoothelin | 1:400 | 1:100 | 1:1000 | Abcam, ab204305 |
| Anti-SM22 | 1:400 | 1:100 | 1:1000 | Abcam, ab89989 |
| Anti- CD31 | 1:100 | - | - | Abclonal, A0378 |
| Anti-Vimentin | 1:1000 | - | - | Cell Signalling, 57415 |
| Anti-FSP1 | 1:400 | 1:100 | - | Abcam, ab68124 |
| Anti-α-actin Alexa fluor 488 | - | 1:100 | - | Abcam, ab197240 |
| Anti-GAPDH | - | - | 1:10000 | Sigma, G9545 |
| **Secondary antibodies** | | | | |
| Anti-Rabbit IgG | - | - | 1:10000 | Cell Signalling 7074S |
| Anti-Mouse IgG | - | - | 1:2000 | Cell Signalling 7076S |
| Anti-Goat IgG | - | - | 1:2000 | Biorad 5160-2504 |
| Alexa Fluor 488 goat anti-mouse IgG | 1:400 | 1:400 | - | ThermoFisher Scientific, A11029 |
| Alexa Fluor 594 goat anti-mouse, IgG | 1:400 | 1:400 | - | ThermoFisher Scientific, A11032 |
| Alexa Fluor 488 donkey anti-goat IgG | 1:400 | 1:400 | - | ThermoFisher Scientific, A11055 |
| Alexa Fluor 594 goat anti-rabbit IgG | 1:400 | 1:400 | - | ThermoFisher Scientific, A11037 |
| Alexa Fluor 488 goat anti-rabbit IgG | 1:400 | - |  | ThermoFisher Scientific, A11034 |

**Table 4.** Rt commercial kit and oligonucleotides kit

| **Rt kit** | **Composed by** | **Reaction volume** |
| --- | --- | --- |
| iScript cDNA Synthesis Kit | Free RNA-ase water | 4 µl |
|  | 5x reverse-transcription  reaction mix | 1 µl |
|  | iScript reverse transcriptase | Variable |
|  | cDNA | Variable |
| **Oligonucleotides** | **Sequence** | **Reference** |
| α-actin Forward | 5'-CAA GTG ATC ACC ATC GGA AAT G-3' | Sigma |
| α-actin Reverse | 5'-GAC TCC ATC CCG ATG AAG GA- 3' | Sigma |
| SM22 Forward | 5'-CAA GCT GGT GAA CAG CCT GTA C-3' | Sigma |
| SM22 Reverse | 5'-GAC CAT GGA GGG TGG GTT CT-3' | Sigma |
| Calponin Forward | 5'-TGA AGC CCC ACG ACA TTT TT-3' | Sigma |
| Calponin Reverse | 5'-GGG TGG ACT GCA CCT GTG TA-3' | Sigma |
| Smoothelin B Forward | 5'-CAGCTGGAGTGATGGGATGGCCTT -3' | Sigma |
| GAPDH Forward | 5’-ATGGGAGCTGGTCATCAAC-3’ | Sigma |
| GAPDH Reverse | 5’-TTGCTGACAATCTTGAGGGA-3’ | Sigma |
